# Accurately adjusted phenothiazine conformations: reversible conformation transformation at room temperature and self-recoverable stimuli-responsive phosphorescence

**DOI:** 10.1038/s41377-024-01716-7

**Published:** 2025-02-26

**Authors:** Yuan Gao, Wentao Yuan, Yuexin Li, Arui Huang, Yuanyuan Fang, Aisen Li, Kai Wang, Bo Zou, Qianqian Li, Zhen Li

**Affiliations:** 1 Hubei Key Lab on Organic and Polymeric Opto-Electronic Materials, Department of Chemistry, Wuhan University, Wuhan, 430072 China; 2 State Key Laboratory of Superhard Materials, College of Physics, Jilin University, Changchun, 130012 China; 3 School of Physical Science and Information Technology, Liaocheng University, Liaocheng, 252059 China; 4 TaiKang Center for Life and Medical Sciences, Wuhan University, Wuhan, 430072 China

**Keywords:** Optical materials and structures, Optical physics

## Abstract

Conformational flexibility is essential to the stimuli-responsive property of organic materials, but achieving the reversible molecular transformation is still challenging in functional materials for the high energy barriers and restriction by intermolecular interactions. Herein, through the incorporation of various steric hindrances into phenothiazine derivatives with different positions and quantities to tune the molecular conformations by adjustable repulsive forces, the folded angles gradually changed from 180° to 90° in 17 compounds. When the angle located at 112° with moderated steric effect, dynamic and reversible transformation of conformations under mechanical force has been achieved for the low energy barriers and mutually regulated molecular motions, resulting in both self-recoverable and stimuli-responsive phosphorescence properties for the first time. It opened up a new way to realize the self-recovery property of organic materials, which can facilitate the multi-functional property of smart materials with the opened avenue for other fields with inspiration.

## Abstract (web summary)

Stimuli-responsive phosphorescence with self-recovery property has been achieved for the first time by precisely modulating molecular conformations of phenothiazine moieties.

## Graphical abstract

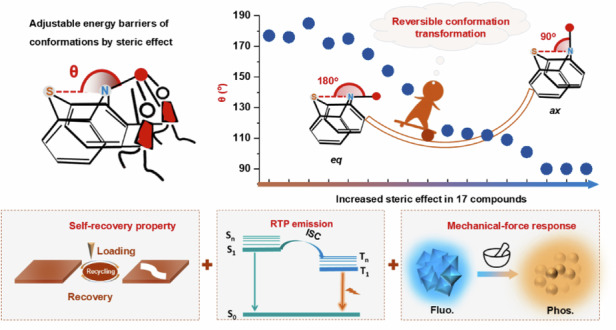

## Introduction

Transformations of molecular conformations play a crucial role in molecular functions and chemical transformations^1^, such as the importance of conformational dynamics in enzyme catalysis^2–4^. In material science, molecular conformations not only influence the functions at isolated states, but also the aggregated structures for the steric and electronic effect, usually resulting in the large difference in material functions by modulated conformations^5–7^, for example, stimulus-responsive organic room-temperature phosphorescence (RTP)^8–15^. It was mainly due to the high sensitivity of emission performance derived from the intersystem crossing process and the stabilization of excited triplet states towards molecular conformations^16–22^. However, it is very difficult to precisely tune the molecular conformation at the stage of molecular design, and there is a lack of a summary of the relationship between molecular conformations and material performance. As to dynamic conformation and the further investigation of reversible transformations, it is a pity that no reports are concerned.

**Fig. 1:**
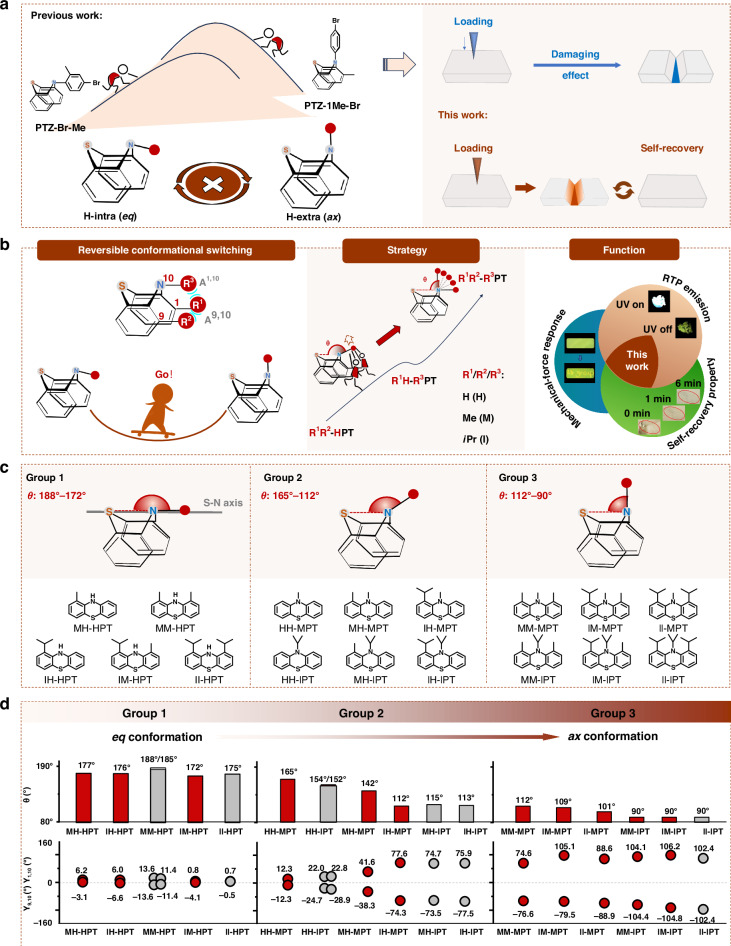
Molecular design for stimuli-responsive phosphorescence materials with rapid self-recovery property. **a** Schematic representation of phenothiazine derivatives with the irreversible process between *ax* and *eq* conformations in the previous study, and the self-recovery stimulus-responsive property achieved in this work. **b** Schematic representation of the self-recovering process of phenothiazine derivatives with reversible switching of *ax* and *eq* conformations in this work. Schematic diagram of different conformations of phenothiazine derivatives by the introduction of various substituents at 1-(R^1^), 9-(R^2^), and 10-(R^3^) positions of phenothiazine skeleton with adjustable steric effect. Combination of RTP emission, self-recovery property, and mechanical-force response by adjustable molecular conformations with muti-functions. **c** Molecular structures of phenothiazine derivatives in Groups 1–3, which were divided by folding angle θ. **d** Dependence relationship among steric hindrance of various substituents, folding angle θ, and torsion angle γ^1,10^/γ^9,10^ of phenothiazine derivatives in Group 1–3. These parameters with red labels were extracted from single-crystal structures of phenothiazine derivatives, which were collected in a Bruker Smart Apex CCD diffractometer. These parameters with grey labels were from optimized molecular conformations at the PBE1PBE/def2svp level in the Gaussian 09 program

Phenothiazine, with a functionalization position on the nitrogen atom, possesses two different conformations of H-intra (quasi-equatorial, *eq*) and H-extra (quasi-axial, *ax*) by *N*-inversion or the ring inversion, similar to that of cyclohexanes, offering its derivatives with totally different emission properties for varied conformations^23–27^. For example, PTZ-1Me-Br with *ax*-conformation demonstrates persistent RTP at aggregated state, but PTZ-Br-Me with *eq*-conformation does not (Fig. 1a)^28^. However, the two compounds could not change to the opposite conformation because of the large energy barriers caused by the presence of the large steric effect. Even though, the minor structural difference between PTZ-1Me-Br and PTZ-Br-Me indicates the possible room for tuning of molecular conformations with rational molecular design, and the key point should be the accurate modification of the steric effect.

Prompted by the above considerations, in this work, seventeen phenothiazine derivatives with different numbers (from 1 to 3) and sizes of substitutions (from the hydrogen atom (H) to methyl (M), then to isopropyl (I)) in the 1 (R^1^), 9 (R^2^), and 10 (R^3^) positions of phenothiazine skeleton have been designed and synthesized to modulate the energy barriers of conformation isomers with tunable steric effect (Fig. 1b). The folded angles θ can cover the whole region between *eq* (180°) and *ax* (90°) conformations by the adjustable molecular conformations (Fig. 1c). Accordingly, the large energy gap between the totally different *eq*- and *ax*-conformations sharply decreased, thus facilitating their possible conversion. Really, among them, IH-MPT with the minimized energy barriers demonstrated the self-recovery fluorescence-phosphorescence switching under mechanical force at room temperature for the first time, in addition to the reported self-recovering mechanochromic fluorescence ones^29–33^. The cyclic process can be repeated over 50 times, beneficial to practical applications in recyclable thermal printing, anti-counterfeiting, and information encryption. Moreover, the internal mechanism of self-recovery RTP property by adjustable molecular conformations has been systematically investigated by crystal analysis, in-situ emission property under mechanical force and high hydrostatic pressure, with the aid of theory calculations. It afforded an efficient way to achieve the combination of RTP emission, self-recovery, and force-responsive property by the changeable molecular conformations with largely decreased energy barriers, which is valuable to the development of smart RTP property with wide applications. Thus, this systematical research demonstrates the accurate tuning of molecular conformations by rational molecular design, inspiring other functional materials related to the conformation including medicines.

## Results

### Molecular design and characterization

The transformation between *eq* and *ax* conformations of phenothiazine is usually irreversible at aggregated states without the aid of solvent fuming and heat, because of the relatively high energy barrier^26^. For instance, PTZ-1Me-Br and PTZ-Br-Me as mentioned above, the only difference point between them is the substituted position of the methyl group, which induces the different steric effects. Thus, if the steric effect was adjusted more carefully, it is possible to tune the resultant conformation accurately. From this point, firstly, the phenyl substituents linked to the nitrogen atom of phenothiazine should be removed, and alternatively, other substituents with small sizes should be introduced instead. Accordingly, seventeen phenothiazine derivatives R^1^R^2^-R^3^PT (R^1^/R^2^/R^3^ = -H, -Me, -*i*Pr) with different numbers and sizes of alkyl chains have been designed and synthesized by Buchwald-Hartwig coupling of aniline and aryl bromides (Supplementary Scheme S1 in the supplementary information), cyclization reaction, and nucleophilic substitution. All the target molecules have been characterized by ^1^H NMR, ^13^C NMR, mass spectrometry, elemental analysis, and HPLC (Supplementary Figs. S1–S38 in the supplementary information).

Organic crystals of these phenothiazine derivatives were cultivated by slow solvent evaporation from dichloromethane/methanol solutions except for MH-IPT and IH-IPT with semi-solid states (Supplementary Tables S1–S3 in the supplementary information). The folded angle (θ) along R^3^-N_10_-S and dihedral angle (α) of two phenyl units in phenothiazine moieties are employed to analyze molecular conformations at crystal states in detail (Fig. 1d and Supplementary Fig. S39 in the supplementary information). Meanwhile, the spatial repulsions A^1,10^/A^9,10^ by substituents in these sites can be expressed by the torsion angle γ^1,10^/γ^9,10^ (determined by C_1_, C_11_, N_10_, and R^3^), respectively. Accordingly, θ angles varied from 180° to 90° with the adjustable steric effect, corresponding to the conversion from *eq* to *ax* conformation (Supplementary Fig. S40 in the supplementary information), while α angles only exhibited slight changes from 165° to 130° (Supplementary Fig. S41 in the supplementary information). It indicated that these substituents with various steric effects mainly affect molecular conformations along the S-N axis, mainly due to the large spatial repulsion among them at 1-, 9-, and 10-positions of phenothiazine. To carefully analyze the detailed changes in molecular conformations, these phenothiazine derivatives were divided into three groups with varied steric effects:

1. Group 1 (*eq* conformations): phenothiazine derivatives (MH-HPT, IH-HPT, MM-HPT, IM-HPT and II-HPT) bearing one substituent at 1-position (R^1^) or two substituents at 1-position (R^1^) and 9-position (R^2^), which demonstrated little steric effect with the small torsion angles γ^1,10^/γ^9,10^ (0.7° ~ 13.6°/−0.5 ~ −13.6°). Accordingly, they demonstrated the *eq* conformations with θ angles in the region of 188° ~ 172°.
2. Group 2 (transitional conformations): phenothiazine derivatives (HH-MPT, HH-IPT, MH-MPT, IH-MPT, MH-IPT, and IH-IPT) with one substituent located at 10-position (R^3^), or two substituents at 10-position (R^3^) and 1-position (R^1^). They exhibited transitional conformations between *eq* and *ax* ones, with θ angles ranging from 165° to 112°. The torsion angles γ^1,10^/γ^9,10^ increased from 12.3° ~ 22.8°/−12.3° ~ −28.9° to 41.6° ~ 77.6°/−38.3° ~ −77.5° with the increased number of substituents.
3. Group 3 (*ax* conformation): phenothiazine derivatives (MM-MPT, IM-MPT, II-MPT, MM-IPT, IM-IPT, and II-IPT) with three substituents located at 1,9,10-positions. θ angles of these molecules were about 90° as *ax* conformation, mainly due to the large repulsion forces among the three substituents, as proved by the large torsion angles γ^1,10^/γ^9,10^ of about 106°/-104°.

### Photophysical property

Thus, the molecular conformation of phenothiazine can be well-tuned by the various steric effects of substituents with different sizes and numbers, which can cover the whole region ranging from *eq* to *ax* ones. The incorporation of substituents with various steric effects into phenothiazine derivatives usually resulted in the blue-shifted absorption spectra in dilute DCM solution (10 *µ*M) (Fig. S42 in the supplementary information), compared to that of phenothiazine skeleton (HH-HPT), which was mainly related to the twisted molecular conformations. In detail, for Group 1 with small steric effects, nearly no blue shift can be observed, indicating similar conformations to that of phenothiazine itself. Once the steric effect increased in Group 2, the onset absorption wavelength of the corresponding luminogens demonstrated about 60 nm blue-shift, and they can be enlarged to about 73 nm with the further increased steric effect by three substituents in Group 3. These indicated the weakened conjugated effect by substituents with increased sizes and numbers. Accordingly, the photoluminescence spectra of phenothiazine derivatives in Group 1 exhibited similar emission wavelengths to that of phenothiazine itself (411 nm) (Table 1 and Supplementary Fig. S43 in the supplementary information). The obvious blue shifts (about 10 ~ 20 nm) can be detected in Group 3 for the twisted conformations. However, both blue- and red-shifted emissions can be observed in Group 2, possibly due to the conformation changes at excited states in these luminogens with highly asymmetric structures. Once the temperature decreased to 77 K (Supplementary Fig. S44 in the supplementary information), phenothiazine derivatives in Group 1 still kept the similar phosphorescence spectra with that of the phenothiazine skeleton, but the phosphorescence peaks became broadened in Group 2 and Group 3, mainly due to the existence of multiple excited triplet states by the possible changes of molecular conformations.

**Table 1:** Photophysical Property of phenothiazine derivatives with different substituents

Compound		Solution state^[a]^			Crystal^[b]^ or Semi-solid^[c]^ state				Ground state^[d]^		
		${{\mathbf{\lambda }}}_{{\boldsymbol{Abs}}.,{\mathbf{max .}}}^{{\boldsymbol{a}},{\boldsymbol{RT}}}$ (nm)	${{\mathbf{\lambda }}}_{{\boldsymbol{PL}},{\mathbf{max .}}}^{{\boldsymbol{a}},{\boldsymbol{RT}}}$ (nm)	${{\mathbf{\lambda }}}_{{\boldsymbol{P}}{\boldsymbol{hos}}.,{\mathbf{max .}}}^{{\boldsymbol{a}},{\boldsymbol{77K}}}$ (nm)	${{\mathbf{\lambda }}}_{{\boldsymbol{PL}},{\mathbf{max .}}}^{{\boldsymbol{b/c}},{\boldsymbol{RT}}}$ (nm)	${{\mathbf{\lambda }}}_{{\boldsymbol{P}}{\boldsymbol{hos}}.,{\mathbf{max .}}}^{\boldsymbol{b/c},{\boldsymbol{RT}}}$ (nm)	${{\mathbf{\tau }}}_{\boldsymbol{P}{\boldsymbol{hos}}.}^{\boldsymbol{b/c},{\boldsymbol{RT}}}$ (ms)	${\boldsymbol{\Phi }}_{\boldsymbol{{PL}}}^{\boldsymbol{b/c},{\boldsymbol{RT}}}$ (%)	${{\mathbf{\lambda }}}_{\boldsymbol{{PL}},{\mathbf{max .}}}^{\boldsymbol{d},{\boldsymbol{RT}}}$ (nm)	${{\mathbf{\lambda }}}_{\boldsymbol{P}{\boldsymbol{hos}}.,{\mathbf{max }}}^{\boldsymbol{d},{\boldsymbol{RT}}}$ (nm)	${{\mathbf{\tau }}}_{\boldsymbol{P}{\boldsymbol{hos}}.}^{\boldsymbol{d},{\boldsymbol{RT}}}$ (ms)
1	MH-HPT	373	411	507	443	–	–	1.62	444	–	–
	IH-HPT	373	411	507	437	520	0.4	1.23	437	520	0.4
	MM-HPT	373	411	507	448	460	1.0	2.84	447	460	4.1
	IM-HPT	373	411	507	438	506	1.6	3.76	440	506	1.1
	II-HPT	373	411	507	436	503	4.1	3.25	436	503	1.8
2	HH-MPT	313	450	510	427	496	1.1	3.00	430	588	1.0
	HH-IPT	313	412	500	424	520	6.9	1.12	424	540	1.7
	MH-MPT	313	455	517	496	496	1.6	3.27	422	580	3.3
	IH-MPT	313	460	514	494	494	8.0	15.44	550	550	7.0
	MH-IPT	313	348	510	455	455	0.1	3.32	–	–	–
	IH-IPT	313	348	510	437	–	–	6.30	–	–	–
3	MM-MPT	300	402	520	504	504	10.1	8.74	504	504	10.4
	IM-MPT	300	402	530	506	506	19.9	6.92	506	506	17.7
	II-MPT	300	397	535	496	502	30.1	14.79	500	502	31.5
	MM-IPT	300	399	516	505	514	23.3	9.53	505	514	27.6
	IM-IPT	300	393	520	509	520	22.6	13.21	509	520	22.4
	II-IPT	300	392	520	460	509	0.9	12.97	434	500	1.9

^[a]^In DCM solution with a concentration of 10 *μ*M. ^[b]^Crystal state. ^[c]^Semi-solid state. ^[d]^Ground state. *λ*_*Abs.,max*_: the maximum emission wavelength of UV-Vis absorption spectrum. *λ*_*PL,max*_: the maximum emission wavelength of Photoluminescence spectrum (excitation wavelength: 315 nm,^[a]^ 365 nm^[b]/[c]/[d]^). *λ*_*Phos.,max*_: the maximum emission wavelength of phosphorescence spectrum at room temperature (excitation wavelength: 365 nm^[b]/[c]/[d]^). *τ*_*Phos*._: the average lifetime of RTP emission. *Φ*_*PL*_: Photoluminescence quantum yield (excitation wavelength: 365 nm^[b]/[c]^)

**Fig. 2:**
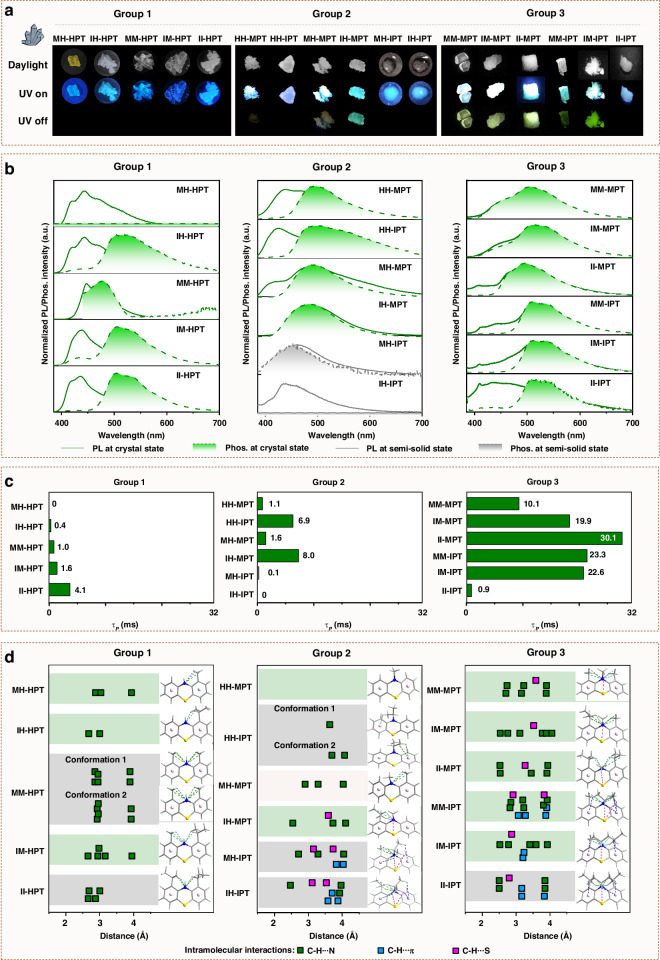
Photophysical property and crystal analysis of phenothiazine derivatives. **a** Photographs of phenothiazine derivatives at crystalline state/semi-solid states under different conditions. **b** Normalized steady-state photoluminescence and phosphorescence spectra of phenothiazine derivatives (Excitation wavelength: 365 nm). **c** The phosphorescence lifetime (*τ*_*p*_) of phenothiazine derivatives at room temperature. **d** The intramolecular interactions of phenothiazine derivatives. The green background meant that the molecular conformations of phenothiazine derivatives were from crystal structures, while the grey background presented these molecular conformations were optimized at PBE1PBE/def2svp level in the Gaussian 09 program

Once these phenothiazine derivatives aggregated into solid states, all of them exhibited extended absorption spectra, compared to those in solution (Supplementary Figs. S45–S46 in the supplementary information), mainly due to the multiple intermolecular interactions at aggregated states. Under UV irradiation (365 nm), bright fluorescence can be observed with the emission colors changing from blue (Group 1) to blue-green/green (Group 2 and Group 3) (Fig. 2a and Supplementary Fig. S47 in the supplementary information). Accordingly, the emission wavelengths of photoluminescence spectra red-shifted from 436 nm to 509 nm with the increased steric effect from Group 1 to Group 3 (Fig. 2b and Table 1). The higher photoluminescence quantum yields were obtained by IH-MPT (Group 2) and II-MPT (Group 3) with 15.44% and 14.79%, respectively (Table 1). After removing UV source, only MH-MPT and IH-MPT with approximate *ax* conformations in Group 2, and MM-MPT, IM-MPT, II-MPT, MM-IPT, and IM-IPT with *ax* conformations in Group 3 demonstrated the naked-eyes observed green/yellow afterglow, indicating that the twisted conformations were beneficial to RTP emission (Fig. 2a). It was mainly due to their varied excited triplet states (T_n_) with distinguished features and energy levels by the adjustable molecular conformations. The smaller energy gaps of *ax* conformations between adjacent T_n_ of phenothiazine derivatives, compared to those of *eq* conformations, can lead to the faster internal conversion (IC) rates of triplet excitons, then restrict the triplet quenching caused by exciton collision in high concentrations^34–36^. Also, the existing forms can affect RTP properties at solid states, MH-IPT and IH-IPT with semi-solid state, and HH-IPT and II-IPT crystals with poor quality demonstrated the extremely weak and invisible RTP, no matter they can form approximative *ax* conformations by theory calculation at PBE1PBE/def2svp level in the Gaussian 09 program^37^. It may be due to the disordered molecular arrangement at aggregated states, which can not efficiently suppress the non-radiative transitions. For phenothiazine derivatives with ordered packing as single crystals, luminogens in Group 3 demonstrated longer phosphorescence lifetimes (10.1 ms ~ 30.1 ms) than those in Group 1 (0 ms ~ 4.1 ms) and Group 2 (1.1 ms ~ 8.0 ms), further confirming the positive effect by multiple substituents with large steric hindrance, and the preferred *ax* conformation of phenothiazine moiety to RTP property (Fig. 2c and Supplementary Fig. S48 in the supplementary information).

### Crystal structure

To systematically investigate the substituent effect on RTP property, the crystal structures of these phenothiazine derivatives were analyzed in detail. Apart from the varied conformations of phenothiazine skeleton from *eq* to *ax* ones by the increased steric effect of substituents as mentioned above, the alkyl substituents can induce multiple intra-/intermolecular interactions, including C-H···π, C-H···N and C-H···S interactions, which can efficiently restrict the possible molecular motions at aggregated states. The intramolecular interactions of these phenothiazine derivatives varied greatly in the three groups with different alkyl substituents. In Group 1, only C-H···N intramolecular interaction can be detected with distances of 2.54 Å ~ 3.84 Å, and quantities ranging from 2 to 6. For phenothiazine derivatives in Group 2 with one substituent (R^3^) at 10-position (HH-R^3^PT), the intramolecular interactions are still weak with only C-H···N interaction. However, once the substituents at 1-position (R^1^) were incorporated, the intramolecular interactions of the resultant luminogens (R^1^H-R^3^PT) strengthened gradually with multiple C-H···π, C-H···N and C-H···S interactions. They can further strengthen with the increased number of substituents to three (Group 3) (Fig. 2d and Supplementary Fig. S49 in the supplementary information). The intermolecular interactions of phenothiazine derivatives in these three Groups with different conformations demonstrated little difference, except for the emerged π-π interactions in MM-MPT, II-MPT, and MM-IPT crystals (Group 3), mainly due to the changeable molecular packing (Supplementary Figs. S50–S51 in the supplementary information). Thus, the improved RTP property from Group 1 to Group 3 was mainly due to the following three points: 1) The conformation conversion from *eq* to *ax* by increased steric effect of substituents, and *ax* conformation is proved as the preferred one, 2) The strong intramolecular interactions by the increased number of substituents, which can restrict molecular motions as non-radiative transitions, beneficial to RTP emission. 3) the π-π coupling at crystal states, which can efficiently stabilize the excited triplet states to prolong RTP emission.

### Mechanical force-stimulus response

**Fig. 3:**
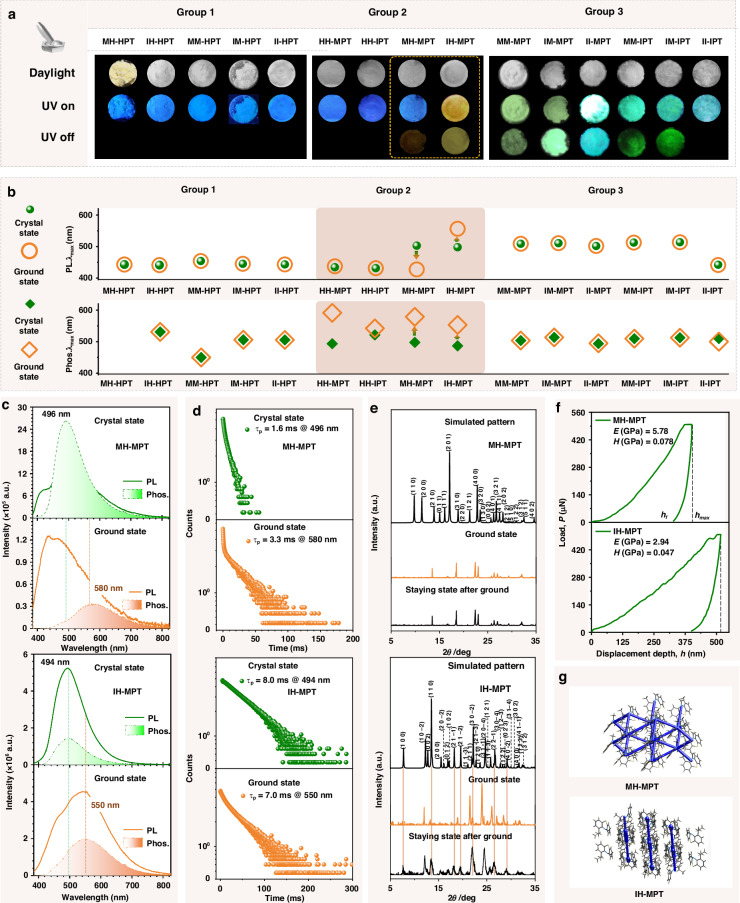
Mechanical-stimulus property of phenothiazine derivatives. **a** Photographs of phenothiazine derivatives at ground state under different conditions. **b** The maximum emission wavelength (λ_max_) of photoluminescence (PL) (top) and room-temperature phosphorescence (RTP) spectra (down) of phenothiazine derivatives at the crystal (green pattern) and ground (orange pattern) states. **c** Photoluminescence (solid line) and phosphorescence spectra (dotted line) of MH-IPT and IH-MPT at the crystal (green) and ground (orange) states. **d** Photoluminescence decay of MH-MPT and IH-MPT at the crystal and ground state. **e** Powder X-ray diffraction (PXRD) patterns of MH-MPT and IH-MPT (the simulated XRD pattern calculated from single-crystal X-ray data with Mercury 2022.2.0) at different states, including crystal state, ground state, and staying state after ground. **f** *P-h* curves of MH-MPT and IH-MPT crystals. **g** Energy frameworks of MH-MPT and IH-MPT crystals

Subsequently, the mechanical-stimulus response of these phenothiazine derivatives with flexible molecular conformations was investigated. As shown in Fig. 3a, the emission colors of organic luminogens in Group 1 and Group 3 demonstrated little changes after being ground, compared to those in crystal state, as further confirmed by their similar photoluminescence spectra at the crystal and ground state (Fig. 3b and Supplementary Fig. S52 in the supplementary information). Also, their RTP property exhibited little difference in phosphorescence emission wavelengths (Fig. 3b and Supplementary Fig. S53 in the supplementary information) and lifetimes (Fig. S54 in the supplementary information). These were mainly due to the maintainable crystalline states after being ground, as proved by the similar XRD patterns of these luminogens at crystal and ground states (Supplementary Fig. S55 in the supplementary information). Interestingly, the phenothiazine derivatives in Group 2 demonstrated the obvious mechanical stimulus-response property. The mechanical stimulus-reponsive property may also be related to the asymmetrical structures in Group 2, which can be easily interfered by external force for the unevenness of electron distribution, leading to structural distortions in bond lengths and angles, as well as electron redistribution. Accordingly, the dynamic RTP property can be obtained for the varied excited triplet states with different energy levels and lifetimes. For HH-MPT and HH-IPT with one substituent at 10-position, the 92 nm and 20 nm red-shifts of phosphorescence spectra under mechanical force can be detected respectively. Once the additional substituents were incorporated into the 1-position of the phenothiazine skeleton, the resultant luminogens MH-MPT and IH-MPT exhibited obvious changes in emission colors after being ground. Accordingly, the blue-shift (74 nm) of photoluminescence spectra, and red-shift (84 nm) of phosphorescence spectra can be detected for MH-MPT from crystal state to ground state (Fig. 3b). An emission peak at 550 nm emerged in the photoluminescence spectra of IH-MPT at the ground state (Fig. 3c), accompanied by the emission colors changing from blue to orange. The corresponding lifetime was measured as 7.0 ms@550 nm (Fig. 3d), which can be considered as phosphorescence emission. It can be further confirmed by the similar phosphorescence emission wavelength (550 nm) of IH-MPT at the ground state. These variations can be related to the possible changes in aggregated structures and molecular conformations under mechanical force. The new crystal faces (2 0 1), (3 0 −1), (1 0 −4), (3 0 −3), and (2 1 3) in XRD pattern can be detected after being ground, while those of MH-MPT show little difference (Fig. 3e).

More interestingly, the emission color (orange) of IH-MPT in ground state can recover to the initial green one rapidly, just being placed in the air. The reversible transformation of IH-MPT toward mechanical stimulus can be further confirmed by the similar XRD patterns. It is the first time to achieve the mechanical force-induced RTP emission with self-recovering property. However, the self-recovery property can not be found in MH-MPT with similar structures, the corresponding emission property can only be recovered by heating or exposure to organic solvents (Supplementary Fig. S56 in the supplementary information). It was mainly related to the different mechanical properties of these crystals and can be partially explained by the nanoindentation experiments and crystal structures (Fig. 3f). The *P-h* curve of the IH-MPT crystal showed that the maximum penetration depth (*h*_*max*_: 516 nm), and the residual depths (*h*_*f*_: 396 nm) are higher than those of MH-MPT (*h*_*max*_: 388 nm, *h*_*f*_: 310 nm). The corresponding elastic modulus (*E*) and hardness (*H*) of IH-MPT (2.94 GPa and 0.047 GPa) were much lower than those of MH-MPT (*E*: 5.78 GPa, *H*: 0.078 GPa). These indicated the more ductile property of IH-MPT crystal, beneficial to the self-recovering process under mechanical stimulus. The flexible property can be further confirmed by the detailed aggregated structures in single crystals. As shown in Fig. 3g, the intermolecular interactions in IH-MPT crystal are formed among aromatics (interaction energy of 22.9 ~ 39.2 kJ·mol^−1^) and alkyl chains (interaction energy of 13.1 ~ 17.1 kJ·mol^−1^) alternatively (Fig. 3g), as confirmed by energy frameworks calculated and visualized on Crystal Explorer version 21.5 at HF/3–21 G level^38^. Accordingly, the slip planes under mechanical force can be formed along the alkyl chain area with weaker interactions, resulting in possible elastic or plastic deformation. However, MH-MPT demonstrated the face-to-edge packing mode with three-dimensional interaction network with similar energies (16.3 ~ 27.2 kJ·mol^−1^), usually leading to a fracture process under mechanical force. The brittle property of MH-MPT crystal may be the main factor for its unrecoverable process.

### Self-recovery property

**Fig. 4:**
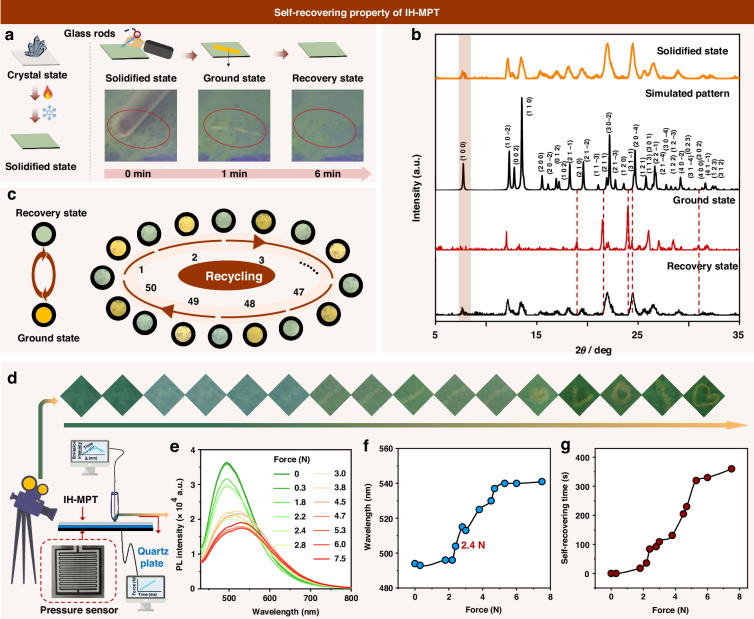
Self-recovering property of IH-MPT. **a** Left: Schematic diagrams of the fabrication process of IH-MPT film by thermal annealing. Right: Schematic diagrams and photographs of IH-MPT film with green-to-orange emission switching and self-recovery property after being scratched. **b** Powder X-ray diffraction (PXRD) patterns IH-MPT (the simulated XRD pattern calculated from single-crystal X-ray data with Mercury 2022.2.0) under different states, including solidified state, ground state and recovery state. **c** The recyclable property of IH-MPT film with green-to-orange emission switching. **d** Experimental setup for detection of the quantitative relationship among pressure force, emission spectra, and self-recovering time, and the emission image of IH-MPT under different forces. **e** The photoluminescence (PL) spectra of IH-MPT under different forces. **f** The quantitative relationship between pressure force and the maximum emission (*λ*_*em*,max_) wavelength. **g** The quantitative relationship between pressure force and self-recovering time

To further investigate the stimuli-responsive RTP with rapid self-recovery property and promote the related applications, IH-MPT film was fabricated by thermal annealing process as the solidified state (Fig. 4a). The corresponding photoluminescence spectra and PXRD pattern were similar to those at crystal state (Fig. 4b), indicating the thermal annealing process has nearly no effect on microcrystalline structure. Once the surface of the IH-MPT film was scratched by a glass rod, the generated scratch demonstrated an obvious orange afterglow (similar to the emission at ground state), which recovered to green emission after 6 min (Fig. 4a and Supplementary Video S1 in the supplementary information). Meanwhile, the scratch disappeared, and the film became smooth rapidly (Supplementary Fig. S57 and Supplementary Video S2 in the supplementary information).

Moreover, the green-to-orange emission switching with the self-recovering property under mechanical stimulus can be repeated more than 50 times (Supplementary Video S3 in the supplementary information), indicating good recyclability (Fig. 4c). Subsequently, the quantitative investigation was conducted by in-situ monitoring of the strength of mechanical force and photoluminescence spectra of IH-MPT at solidified states (Fig. 4d). With the pressure increased gradually from 0 N to 7.5 N (Fig. 4e), the emission wavelengths exhibited the red-shifts from 494 nm to 541 nm. Even the pressure was as low as 2.4 N, a 10 nm red-shift can be detected with the obvious variation of emission colors (Fig. 4f). It indicated that the tiny force can enhance RTP emission, demonstrating the highly sensitive RTP response toward pressure. Moreover, the self-recovering time exhibited a positive correlation with the strength of pressure, which can be as short as 84 s under the pressure of 2.4 N (Fig. 4g), indicating the rapid self-recovering process with sensitive RTP response.

Also, the self-recovery speed is highly related to the temperature of IH-MPT film. The self-recovery time of the “WH” patterns decreased gradually with the increased temperature, in detail, it was about 720 s at 273 K, then decreased to 300 s at 298 K, then to 5 s at 308 K, finally to 0.1 s at 323 K (Supplementary Fig. S58 in the supplementary information). This phenomenon is mainly due to the accelerated molecular motions by thermal effect, benefiting the transformation of molecular conformations as the driving force of self-recovery property. Also, it may be related to the temperature-sensitive property of phosphorescence emission, which disappeared rapidly for the severe non-radiative transitions with the increased temperature.

Additionally, differential scanning calorimetry (DSC) analysis of MH-MPT and IH-MPT was performed by a second heating scan after cooling the melted sample from the first scan to a low temperature^39–41^. The low glass transition temperatures (*T*_g_) are observed at −25.5 °C (MH-MPT) and −25.6 °C (IH-MPT), respectively, which are beneficial to the thermal motions of these luminogens at room temperature (Supplementary Fig. S59 in the supplementary information). The cold-crystallization temperature (*T*_c_) did not occur in both MH-MPT and IH-MPT at crystal or solidified states. Thus, the self-recovery property of IH-MPT should be related to the low glass transition temperature, which could favor molecular motions at room temperature.

**Fig. 5:**
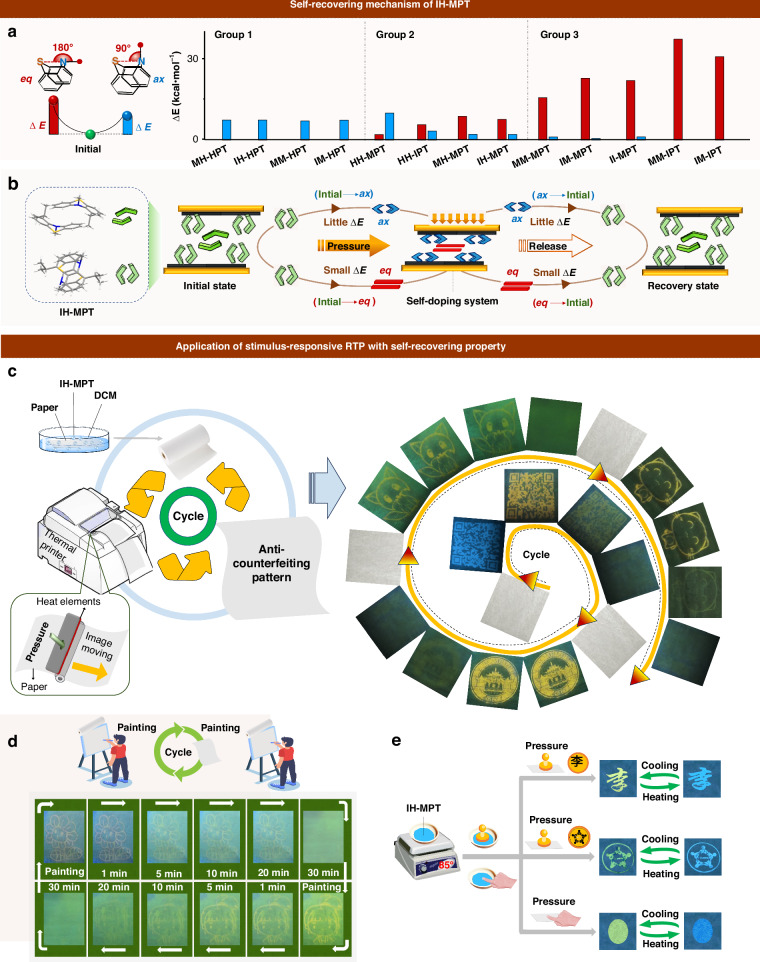
Mechanism and application of mechanical stimulus-responsive IH-MPT film with self-recovering property. **a** Potential surface scanning for phenothiazine derivatives with the folding angle θ ranging from 90° to 180°, ΔE presented the energy gap between *ax*/*eq* conformation and initial one. **b** Schematic representation of bending and/or planarization of IH-MPT in single crystal under pressure and release process. **c** Recycle thermal printing of various patterns by the same paper. **d** Recycle writing and drawing on the same paper to generate different information. **e** Anti-counterfeiting patterns by simple pressing, which demonstrated emission response toward heat and force with recyclable properties

To deeply understand the internal mechanism, the possible conformation changes of phenothiazine skeleton with folded angle θ ranging from 180° (*eq*) to 90° (*ax*) are simulated by potential surface scanning (Fig. 5a). The energy barriers (ΔE) from initial conformations (extracted from crystal structures) to *ax* ones (0.69 kcal·mol^−1^ ~ 7.48 kcal·mol^−1^) are much lower than those with the conversion to *eq* ones (2.08 kcal·mol^−1^ ~ 37.66 kcal·mol^−1^) (Supplementary Tables S4–S6 in the supplementary information), indicating the transformation to *ax* one may be easier under external force in most cases. Among them, phenothiazine derivatives in Group 2 demonstrated the lower barriers (ΔE) of conformation conversions for both initial→*ax* and initial→*eq* processes, which should be related to the moderate steric effect and slightly twisted conformations at initial states. It may result in the high sensitivity of conformation transformation toward external stimuli and the rapid reversible process with lower barriers.

Especially for IH-MPT in Group 2, the column-like molecular packing can be found in the corresponding crystal structure. There were alternative dimer models with perpendicular arrangements in each column, and the two phenothiazine moieties in each dimer demonstrated the face-to-face stacking mode (Fig. 5b). Under mechanical force, the bending and/or planarization process of phenothiazine conformation can occur, since the pressures upon dimer models were from perpendicular directions (Fig. 5b). Thus, both the *ax* and *eq* conformations of the phenothiazine skeleton can be formed at the ground state. Since the smaller energy barrier of initial→*ax* than that of initial→*eq* conformation, more *ax* conformation can be obtained under mechanical force as the host, together with the less *eq* ones as the guest. Thus, the promoted RTP effect with prolonged lifetime and red-shifted emission can be achieved by the resultant self-doping systems. It was mainly due to the rigid matrix of the host, and the possible Förster resonance energy transfer (FRET) from host to guest. Accordingly, it can recover to the initial state rapidly for the lower energy barriers of conformation transformation for IH-MPT, resulting in the self-recovery process with emission switching.

Moreover, the almost linear relationship between force and emission wavelength has been formed by IH-MPT when the strengths of mechanical force were in the region of 2 N ~ 6 N, accompanied by the similar phenomenon of the relation between force and self-recovering time. It is mainly due to the adjustable ratios of *eq* and *ax* conformations of phenothiazine moieties under mechanical force. For the relationship between the maximum emission wavelength and mechanical force, the red-shifted emission wavelengths under mechanical force are due to the generation of RTP under mechanical force. Since the initial conformation of IH-MPT is the transition one between *eq* and *ax* conformations, and the energy barriers of initial→*ax* is lower than that of initial→*eq*, the *ax* conformations are easily formed under mechanical force with low strength, and the ratio of *eq* conformations may increase with the increased strength of mechanical force. Accordingly, the RTP property was promoted with the formation of self-doping system by the mixture of *eq* and *ax* conformations, resulting in the gradually red-shifted emission wavelength with decreased fluorescence emission and increased phosphorescence emission at the same time. For the relationship between the self-recovery time and mechanical force, the increased strength of mechanical force can promote the formation of *eq* conformations, thus, the self-recovery time will increase for the higher energy barrier of *eq*→initial transformation.

Also, the conformation transformation of IH-MPT under pressure can be confirmed by in situ high-pressure properties with pressure ranging from 0 Gpa to 36.0 Gpa (Supplementary Figs. S60–S64 in the supplementary information). Therefore, stimuli-responsive phosphorescence materials with rapid self-recovery property achieved by IH-MPT were mainly due to the following factors: (1) lower energy barriers of conformation transformation adjusted subtly by the intelligently introduced substituents. It can realize the reversible transformation among original state, *ax*, and *eq* ones, promoting the self-recovery property by rapid molecular motions with determined directions; (2) Special molecular arrangement. The column-like packing can be favorable to form the slip planes under mechanical force, which can enhance the impact ductility of organic crystals, beneficial to the self-recovery property. Moreover, the perpendicular dimer models in each column facilitate the formation of self-doping system with the mixture of *ax* and *eq* conformations under mechanical force, resulting in the improved RTP property by the efficient energy transfer.

### Applications

The excellent mechanical force-induced RTP emission of IH-MPT with the self-recovering property can be applied to the recyclable information presentation conveniently. As shown in Fig. 5c, the filter paper was firstly immersed into the IH-MPT solution (concentration: 8 mg·mL^−1^), and then dried to fabricate the recyclable matrix for printing and painting. Accordingly, various patterns, including the QR code, the small fox, the Wuhan University Logo, and the kitten pattern, can be presented on the same paper with multiple cycles of printing and self-recovering process by simply being placed at room temperature for 28 min. Also, writing and drawing with a glass rod can realize the various patterns on the same paper with nearly no interference (Fig. 5d), demonstrating excellent recyclability. Furthermore, the self-evanescent pattern of the recovery process can be applied to the technology of maintaining secrecy. For the confidential information by writing on printing paper with IH-MPT, it can automatically delete without any trace after being read within 28 min to ensure the security. Also, the patterns by stamping, including Chinese characters, Logos, and fingerprint, can be obtained with IH-MPT as an inkpad, which demonstrated highly recyclable temperature-dependent emission for generation of RTP emission under pressing (Fig. 5e).

## Discussion

In summary, stimuli-responsive phosphorescence materials with rapid self-recovery properties have been achieved by phenothiazine derivatives with adjustable molecular conformations and aggregated structures. The tunable steric effect by substituents with different sizes and numbers has been proved as the efficient strategy, to control the molecular conformations and energy barriers. IH-MPT with moderate steric effect can realize the reversible conformation transformation among original state, *ax* and *eq* ones as the preferred conformations for the lower energy barriers. Thus, the self-recovery property by rapid molecular motions with determined directions has been achieved. Moreover, the stimuli-responsive property can be realized by the promoted RTP emission for the formation of self-doping system with the mixture of *ax* and *eq* conformations under mechanical force, contributing to the collaboration of RTP, self-recovery property and stimulus-response into a single-component system. To further accelerate the self-recovery rates, the decreased energy barriers and the weak intermolecular interactions with loose molecular packing may be the preferred factors to the conformational transitions at aggregated states. It paves an efficient way to tune the molecular conformations to explore the smart emission materials with high sensitivity toward external stimuli, superior processability and recyclability, promoting their wide applications for the added high-safety and convenience.

## Materials and methods

### Materials characterization

^1^H and ^13^C NMR spectra were recorded on a Bruker Avance III HD 400 MHz using tetramethylsilane (TMS, δ = 0 ppm) as the internal standard. Elemental analyses (EA) were performed by a Perkin-Elmer microanalyzer. Mass spectra (MS) were measured on a ZAB 3F-HF mass. High-performance liquid chromatography (HPLC) was conducted on Agilent 1100. UV-vis absorption spectra were conducted on a Shimadzu UV-2550 spectrometer. UV-vis absorption spectra were conducted on an FLS980 spectrometer. Fluorescence and phosphorescence spectra, quantum yields, and lifetimes were determined with a Hitachi F-4700 fluorescence spectrophotometer or FLS980 spectrometer. The steady-state emission and persistent phosphorescence emission of IH-MPT were carried out by an Ocean Optics E65 Pro spectrometer with a 254 nm/365 nm Handheld UV lamp as the excitation source. Nanoindentation experiments were performed by a TI 950 TriboIndenter system. The photos and videos were taken by Nikon Z9 and Xiaomi 12Pro.

### Single crystal X-ray diffraction (XRD) data

Single crystals of phenothiazine derivatives were cultivated by slow solvent evaporation from its dichloromethane/methanol solutions at room temperature. The single-crystal X-ray diffraction data of phenothiazine derivatives were collected in a Bruker Smart Apex CCD diffractometer. D8 Advanced (Bruker) recorded the powder X-ray diffraction patterns using Cu-Kα radiation from 5° to 65°.

### Pressure-dependent experiments of crystals

In-situ UV-vis absorption and PL micrographs of the samples were obtained using a camera (Canon Eos 5D mark II) equipped with a microscope (Ecilipse TI-U, Nikon). The camera can record the photographs under the same conditions, including exposure time and intensity. Absorption spectra were measured in the exciton absorption band region using a Deuterium-Halogen light source, and the excitation source a 355 nm line of a UV DPSS laser with the power of 10 mW was used for PL measurements. The fiber spectrometer is an Ocean Optics QE65000 spectrometer. In-situ high-pressure Raman spectra were recorded using a spectrometer equipped with liquid nitrogen-cooled CCD (iHR 550, Symphony II, Horiba Jobin Yvon). A 785 nm diode laser was utilized to excite the sample, and the output power was 10 mW. The resolution of the system was about 1 cm^−1^. All of the high-pressure experiments were conducted at room temperature.

### Nanoindentation experiments

An instrumented nanoindentation test was conducted on MH-MPT and IH-MPT preferred crystal faces, employing a Berkovich diamond indenter (TI 950 Triboindenter, at 500 µN load) to measure the Young’s modulus and the hardness of each component.

### In-situ detection experiments of pressure and emission spectra

A quartz plate was placed on a pressure sensor, such that the force was stressed uniformly, which could eliminate the difference in pressure response caused by different positions of force stimulation on the pressure sensor matrix. A filter paper was placed on the quartz plate, and the IH-MPT at solidified state was placed on the filter paper. Subsequently, the different patterns could be written by glass rod on the filter paper with IH-MPT under different pressures. The strength of pressure could be read by the pressure sensor, and the emission intensity could be detected by an Ocean Optics QE65000 spectrometer.

### Theory calculations

TD-DFT calculations were performed on the Gaussian 09 program (Revision D01). The potential energy of each molecular conformation with folded angle θ in the region of 90°–180° was obtained using Becke’s three-parameter exchange function, along with the Lee Yang Parr’s correlation functional (B3LYP), using 6–31 G (d) basis sets based on the structure extracted from a single crystal. MM-HPT, II-HPT, MH-IPT, IH-IPT, and II-IPT were optimized at PBE1PBE/def2svp level in the Gaussian 09 program. Energy frameworks of MH-MPT and IH-MPT were calculated on Crystal Explorer version 21.5 using the HF functional with 3–21 G.

## Supplementary information

SI

Video S1

Video S2

Video S3

Crystal informations

copyright

## Data Availability

The data that support the findings detailed in this study are available in the article and its supplemental information or from the corresponding authors upon reasonable request.
