# Supplementary material for: Accurately adjusted phenothiazine conformations: reversible conformation transformation at room temperature and self-recoverable stimuli-responsive phosphorescence
Source: Light Sci Appl. 2025 Feb 26;14:99. doi: 10.1038/s41377-024-01716-7 (PMC11862005; doi:10.1038/s41377-024-01716-7)
Supplement: Supplementary file 1 — SI [file 41377_2024_1716_MOESM1_ESM.doc]

**Supplementary Information for**

Accurately Adjusted Phenothiazine Conformations: Reversible Conformation Transformation at Room Temperature and Self-Recoverable Stimuli-responsive Phosphorescence

**Yuan Gao,^1^ Wentao Yuan,^1^ Yuexin Li,^1^ Arui Huang,^1^ Yuanyuan Fang,^3^ Aisen Li,^4^ Kai Wang,^3^ Bo Zou,^3^ Qianqian Li*,^1^ and Zhen Li^1,2^**

^1^Hubei Key Lab on Organic and Polymeric Opto-Electronic Materials, Department of Chemistry, Wuhan University, Wuhan 430072, China

E-mail: liqianiqian@whu.edu.cn

^2^TaiKang Center for Life and Medical Sciences, Wuhan University, Wuhan 430072, China

^3^State Key Laboratory of Superhard Materials, College of Physics, Jilin University, Changchun 130012, China

^4^School of Physical Science and Information Technology, Liaocheng University, Liaocheng 252059, China

**S1 - General experimental details**

**Materials**

Aniline derivatives, bromobenzene derivatives, sodium *tert*-butoxide, 1,1ʹ-bis(diphenylphosphino)ferrocene-palladium (II) dichloride dichloromethane complex, 1,2-dichlorobenzene， anhydrous *N,N*-dimethylformamide (DMF), iodomethane, and 2-bromopropane were purchased from Energy Chemical, and used directly without purification. Phenothiazine, sodium hydride (60% dispersion in mineral oil), sulfur, iodine, and magnesium sulfate were obtained from Aladdin Industrial. Toluene was distilled over CaH_2_ and stored under N_2_ atmosphere. Phenothiazine (HH-HPT) was purified and characterized by elemental analysis and HPLC. MH-DPA, IH-DPA, MM-DPA, and IM-DPA were synthesized according to our previous work.^1^

**Materials characterization**

^1^H and ^13^C NMR spectra were recorded on a Bruker Avance III HD 400 MHz using tetramethylsilane (TMS; δ = 0 ppm) as the internal standard. Elemental analyses (EA) were performed by a Perkin-Elmer microanalyzer. Mass spectra (MS) were measured on a ZAB 3F-HF mass. High-performance liquid chromatography (HPLC) was conducted on Agilent 1100. UV-vis absorption spectra were conducted on a Shimadzu UV-2550 spectrometer. UV-vis absorption spectra were conducted on an FLS980 spectrometer. Fluorescence and phosphorescence spectra, quantum yields, and lifetimes were determined with a Hitachi F-4700 fluorescence spectrophotometer or FLS980 spectrometer. The steady-state emission and persistent phosphorescence emission of IH-MPT were carried out by an Ocean Optics E65 Pro spectrometer with a 254 nm/365 nm Handheld UV lamp as the excitation source. Nanoindentation experiments were performed by a TI 950 TriboIndenter system. The photos and videos were taken by Nikon Z9 and Xiaomi 12Pro. Differential scanning calorimetry (DSC) curves were obtained on a Mettler-Toledo DSC3 at a heating rate of 10 ^o^C·min^-1^.

**Single crystal X-ray diffraction (XRD) data**

Single crystals of phenothiazine derivatives were cultivated by slow solvent evaporation from its dichloromethane/methanol solutions at room temperature. The single-crystal X-ray diffraction data of phenothiazine derivatives were collected in a Bruker Smart Apex CCD diffractometer. D8 Advanced (Bruker) recorded the powder X-ray diffraction patterns using Cu-Kα radiation from 5° to 65°.

**Pressure-dependent experiments of crystals**

*In situ* UV-vis absorption and PL micrographs of the samples were obtained using a camera (Canon Eos 5D mark II) equipped with a microscope (Ecilipse TI-U, Nikon). The camera can record the photographs under the same conditions, including exposure time and intensity. Absorption spectra were measured in the exciton absorption band region using a Deuterium-Halogen light source, and the excitation source a 355 nm line of a UV DPSS laser with the power of 10 mW was used for PL measurements. The fiber spectrometer is an Ocean Optics QE65000 spectrometer. *In situ* high-pressure Raman spectra were recorded using a spectrometer equipped with liquid nitrogen-cooled CCD (iHR 550, Symphony II, Horiba Jobin Yvon). A 785 nm diode laser was utilized to excite the sample, and the output power was 10 mW. The resolution of the system was about 1 cm^-1^. All of the high-pressure experiments were conducted at room temperature.

**Nanoindentation** **experiments**

An instrumented nanoindentation test was conducted on MH-MPT and IH-MPT preferred crystal faces, employing a Berkovich diamond indenter (TI 950 Triboindenter, at 500 *µ*N load) to measure the Young's modulus and the hardness of each component.^2^

***In-situ* detection experiments of pressure and emission spectra**

A quartz plate was placed on a pressure sensor, such that the force was stressed uniformly, which could eliminate the difference in pressure response caused by different positions of force stimulation on the pressure sensor matrix.^3^ A filter paper was placed on the quartz plate, and the IH-MPT at solidified state was placed on the filter paper. Subsequently, the different patterns could be written by glass rod on the filter paper with IH-MPT under different pressures. The strength of pressure could be read by the pressure sensor, and the emission intensity could be detected by an Ocean Optics QE65000 spectrometer.

**Theory calculations**

TD-DFT calculations were performed on the Gaussian 09 program (Revision D01). The potential energy of each molecular conformation with folded angle θ in the region of 90°-180° was obtained using Becke’s three-parameter exchange function, along with the Lee Yang Parr’s correlation functional (B3LYP), using 6-31G (d) basis sets based on the structure extracted from a single crystal. MM-HPT, II-HPT, MH-IPT, IH-IPT, and II-IPT were optimized at PBE1PBE/def2svp level in the Gaussian 09 program. Energy frameworks of MH-MPT and IH-MPT were calculated on Crystal Explorer version 21.5 using the HF functional with 3-21G.

**S2 - Synthesis and characterization**

**Synthesis and Characterization**


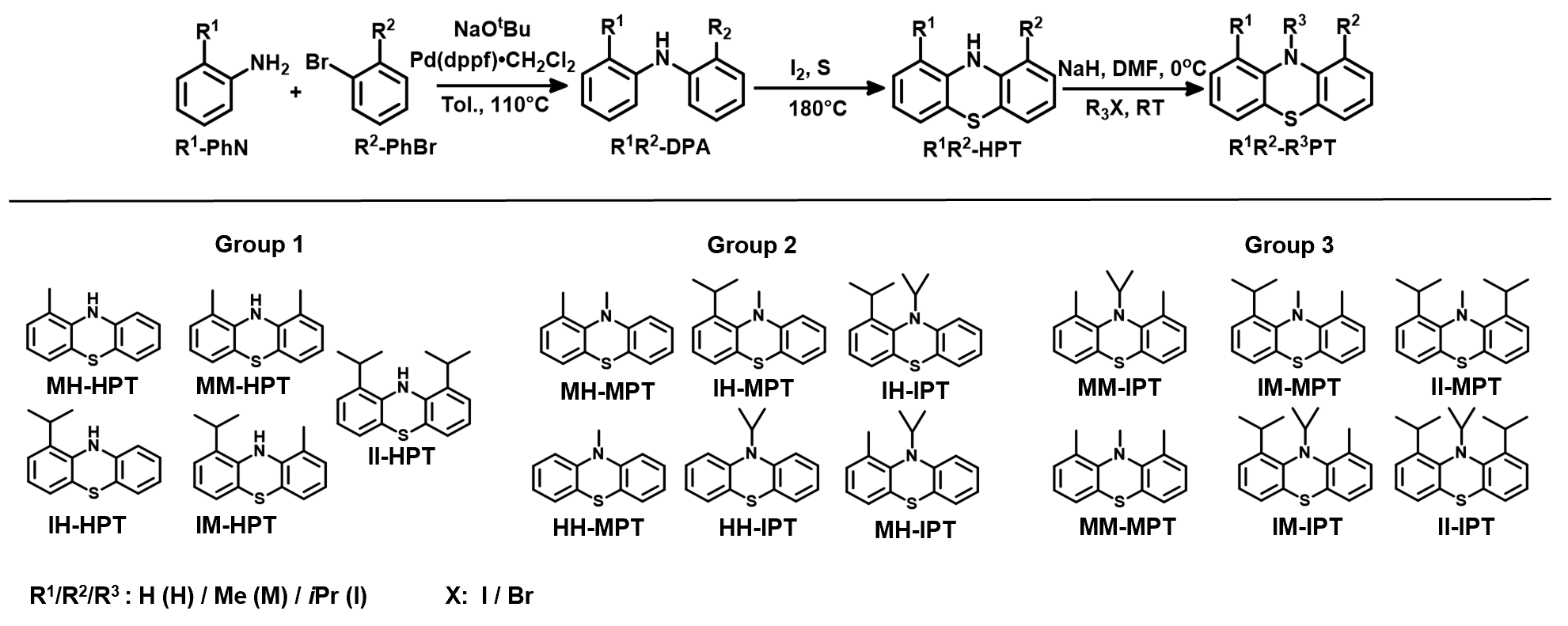


**Scheme S1** The synthetic routes of phenothiazine derivatives.

**Synthesis of R^1^R^2^-DPA: General procedure 1**^1^

Under the atmosphere of nitrogen, to a 250 mL round-bottomed flask equipped was added NaO*^t^*Bu (2.0 eq.) and Pd(dppf)Cl_2_·CH_2_Cl_2_ (0.03 eq.). Dry toluene (100 mL) was then added *via* a syringe, followed by aniline derivatives (1.1 eq.) and bromobenzene derivatives (1.0 eq.). The solution was bubbled with nitrogen for 15 minutes, and the mixture was heated to 110 °C and stirred for 16 h. The toluene was removed from the reaction mixture on a rotary evaporator at 50 °C. The reaction mixture was quenched by diluted hydrochloric acid (1:1) and washed by water, then extracted with CH_2_Cl_2_. The combined organic layer was dried over anhydrous Na_2_SO_4_ and concentrated by rotary evaporation. The compounds R^1^R^2^-DPA were purified by silica gel column chromatography with hexane as eluent.

**Synthesis of R^1^R^2^-HPT: General procedure 2**^4^

Under the atmosphere of nitrogen, to a 100 mL thick-walled pressure-resistant reaction flask was added R_1_R_2_-DPA (1.0 eq.), sulfur (2.0 eq.), and I_2_ (0.028 eq.) and 1,2-dichlorobenzene (9.0 mL). The reaction mixture was then deoxygenated by bubbling with nitrogen for 30 minutes and was then heated to 180°C for 4 hours. After being cooled to room temperature, the crude product was purified by column chromatography on silica gel using petroleum ether as eluent to afford the target product R^1^R^2^-HPT.

**Synthesis of R_1_R_2_-R_3_PT: General procedure 3**^5^

Under the atmosphere of nitrogen, to a 100mL thick-walled pressure-resistant reaction flask was added R^1^R^2^-HPT (1.0 eq.) and anhydrous DMF (10 mL). The solution was immersed in an ice-water bath, and sodium hydride (60 wt.% in mineral oil, 2.2 eq.) was added. After stirring the suspension for 5 min, R^3^X (3.0 eq.) was added to the reaction mixture. The reaction was allowed to warm to room temperature and was stirred for 12 h. The reaction mixture was quenched with ice and extracted in dichloromethane. The combined organic extracts were dried over anhydrous Na_2_SO_4_ and concentrated by rotary evaporation. The crude material was purified by column chromatography over silica gel using hexanes as the eluent, then crystallized from methanol, yielding the product as a white crystalline solid.

**II-DPA:** A yellow oil (2.40 g, 69%). ^1^H NMR (400 MHz, CDCl_3_) δ (ppm): 7.38 (d, *J* = 8.0 Hz, 2H, ArH), 7.22-7.15 (m, 2H, ArH), 7.07 (t, *J* = 7.4 Hz, 4H, ArH), 5.42 (s, 1H, NH), 3.26-3.16 (m, 2H, CH), 1.39 (d, *J* = 8.0 Hz, 12H, CH_3_). ^13^C NMR (100 MHz, CDCl_3_) δ (ppm): 141.47, 138.25, 126.60, 125.86, 121.91, 119.50, 27.89, 22.89.

**MH-HPT:** A yellow solid (1.20 g, 81%). mp: 128-130 ^o^C. ^1^H NMR (400 MHz, DMSO-*d*_6_) δ 7.65 (s, 1H, NH), 7.05-6.97 (m, 2H, ArH), 6.93-6.86 (m, 2H, ArH), 6.77 (t, *J* = 7.3 Hz, 2H, ArH), 6.68 (t, *J* = 7.5 Hz, 1H, ArH), 2.21 (s, 3H, CH_3_). ^13^C NMR (100 MHz, DMSO-*d*_6_) δ 142.66, 140.57, 129.70, 127.74, 126.46, 124.69, 122.87, 122.53, 121.94, 117.70, 116.96, 116.12, 18.11. MS (EI) calcd. for C_13_H_11_NS [M+H]^+^: 213.06; Found: 213.10. Anal. calcd. for C_13_H_11_NS: C, 73.20; H, 5.20; N, 6.57. Found: C, 72.96; H, 5.31; N, 6.87.

**IH-HPT:** A white solid (1.00 g, 83%). mp: 116-118 ^o^C. ^1^H NMR (400 MHz, CDCl_3_) δ 7.06-7.00 (m, 3H, ArH), 6.93 (dd, *J* = 7.7, 1.5 Hz, 1H, ArH), 6.89-6.84 (m, 2H, ArH), 6.65 (dd, *J* = 7.7, 1.2 Hz, 1H, ArH), 6.08 (s, 1H, NH), 2.95 (p, *J* = 6.8 Hz, 1H, CH), 1.31 (d, *J* = 6.8 Hz, 6H, CH_3_). ^13^C NMR (100 MHz, CDCl_3_) δ 142.14, 139.19, 132.02, 127.29, 126.81, 124.83, 123.94, 122.81, 122.43, 119.43, 114.91, 27.58, 22.49. MS (EI) calcd. for C_15_H_15_NS [M+H]^+^: 241.09; Found: 241.15. Anal. calcd. for C_15_H_15_NS: C, 74.65; H, 6.26; N, 5.80. Found: C, 74.23; H, 6.26; N, 6.11.

**HH-MPT:** A white solid (2.40 g, 75%). mp: 92-94 ^o^C. ^1^H NMR (400 MHz, CDCl_3_) δ 7.21-7.09 (m, 4H, ArH), 6.92 (t, *J* = 7.3 Hz, 2H, ArH), 6.80 (d, *J* = 8.0 Hz, 2H, ArH), 3.36 (s, 3H, CH_3_). ^13^C NMR (100 MHz, CDCl_3_) δ 127.44, 127.16, 123.42, 122.47, 114.09, 35.31. MS (EI) calcd. for C_13_H_11_NS [M+H]^+^: 213.06; Found: 213.05. Anal. calcd. for C_13_H_11_NS: C, 73.20; H, 5.20; N, 6.57. Found: C, 73.18; H, 5.17; N, 6.57.

**HH-IPT:** A white solid (3.00 g, 83%). mp: 66-68 ^o^C. ^1^H NMR (400 MHz, CDCl_3_) *δ* (ppm): 7.16-7.11 (m, 4H, ArH), 7.04 (d, *J* = 8.0 Hz, 2H, ArH), 6.94-6.90 (t, *J* = 7.4 Hz, 2H, ArH), 4.35-4.24 (m, 1H, CH), 1.62 (d, *J* = 7.0 Hz, 6H, CH_3_). ^13^C NMR (100 MHz, CDCl_3_) *δ* (ppm): 145.33, 127.32, 126.96, 126.77, 122.61, 118.05, 53.60, 22.41. MS (EI) calcd. for C_15_H_15_NS [M+H]^+^: 241.09; Found: 241.10. Anal. calcd. for C_15_H_15_NS: C, 74.65; H, 6.26; N, 5.80. Found: C, 74.73; H, 6.35; N, 5.96.

**MM-HPT:** A white solid (0.90 g, 68% yield). mp: 124-126 ^o^C. ^1^H NMR (400 MHz, CDCl_3_) *δ* (ppm): 6.90 (d, *J* = 7.2 Hz, 4H, ArH), 6.77 (s, 2H, ArH), 5.83 (s, 1H, NH), 2.25 (s, 6H, CH_3_). ^13^C NMR (100 MHz, CDCl_3_) *δ* (ppm): 141.93, 130.81, 127.50, 126.79, 124.87, 121.35, 118.26, 17.82, 16.89. MS (EI) calcd. for C_14_H_13_NS [M+H]^+^: 227.08; Found: 227.10. Anal. calcd. for C_14_H_13_NS: C, 73.97; H, 5.76; N, 6.16. Found: C, 73.51; H, 5.73; N, 6.47.

**IM-HPT:** A white solid (1.30 g, 84%). mp: 104-106 ^o^C. ^1^H NMR (400 MHz, CDCl_3_) *δ* (ppm): 7.00 (d, *J* = 8.0 Hz, 1H, ArH), 6.91 (t, *J* = 5.4 Hz, 3H, ArH), 6.85 (t, *J* = 7.6 Hz, 1H, ArH), 6.77 (t, *J* = 7.6 Hz, 1H, ArH), 6.15 (s, 1H, NH), 2.99-2.92 (m, 1H, CH), 2.28 (s, 3H, CH_3_), 1.33 (d, *J* = 6.8 Hz, 6H). ^13^C NMR (100 MHz, CDCl_3_) *δ* (ppm): 132.10, 128.86, 123.82, 122.51, 121.65, 27.81, 22.44, 16.90. MS (EI) calcd. for C_16_H_17_NS [M+H]^+^: 255.11; Found: 255.15. Anal. calcd. for C_16_H_17_NS: C, 75.25; H, 6.71; N, 5.48. Found: C, 74.84; H, 6.74; N, 5.82.

**II-HPT:** A white solid (2.00 g, 55%). mp: 84-86 ^o^C. ^1^H NMR (400 MHz, CDCl_3_) *δ* (ppm): 7.07-6.78 (m, 6H, ArH), 6.42 (s, 1H, NH), 2.99-2.92 (m, 1H, CH), 1.33 (d, *J* = 6.8 Hz, 12H, CH_3_). ^13^C NMR (100 MHz, CDCl_3_) *δ* (ppm): 139.18, 132.20, 124.60, 123.79, 122.50, 119.10, 27.79, 22.65. MS (EI) calcd. for C_18_H_21_NS [M+H]^+^: 283.14; Found: 283.20. Anal. calcd. for C_18_H_21_NS: C, 76.28; H, 7.47; N, 4.94. Found: C, 75.90; H, 7.44; N, 5.20.

**MH-MPT:** A white solid (0.90 g, 71%). mp: 44-46 ^o^C. ^1^H NMR (400 MHz, CDCl_3_) δ 7.18-7.10 (m, 2H, ArH), 7.03-6.98 (m, 3H, ArH), 6.95-6.87 (m, 2H, ArH), 3.47 (s, 3H, CH_3_), 2.38 (s, 3H, CH_3_). ^13^C NMR (100 MHz, CDCl_3_) δ 148.54, 144.09, 130.68, 130.29, 130.16, 128.56, 127.11, 126.50, 124.95, 123.69, 123.17, 119.61, 42.17, 20.06. MS (EI) calcd. for C_14_H_13_NS [M+H]^+^: 227.07; Found: 227.05. Anal. calcd. for C_14_H_13_NS: C, 73.97; H, 5.76; N, 6.16. Found: C, 73.70; H, 5.78; N, 6.11.

**IH-MPT:** A white solid (0.90 g, 82%). mp: 84-86 ^o^C.^1^H NMR (400 MHz, CDCl_3_) δ 7.20-7.10 (m, 4H, ArH), 7.04-6.97 (m, 3H, ArH), 3.55-3.48 (m, 1H, CH), 3.33 (s, 3H, CH_3_), 1.24 (s, 6H, CH_3_). ^13^C NMR (100 MHz, CDCl_3_) δ 148.44, 143.33, 132.15, 131.00, 127.23, 126.60, 125.38, 125.37, 124.78, 124.67, 124.05, 122.93, 44.01, 27.99. MS (EI) calcd. for C_16_H_17_NS [M+H]^+^: 255.10; Found: 255.10. Anal. calcd. for C_16_H_17_NS: C, 75.25; H, 6.71; N, 5.48. Found: C, 75.18; H, 6.72; N, 5.45.

**MH-IPT:** A colorless oily (1.30 g, 78%). ^1^H NMR (400 MHz, CDCl_3_) *δ* (ppm): 7.24-7.22 (m, 1H, ArH), 7.18-7.13 (m, 2H, ArH), 7.10-7.05 (m, 3H, ArH), 6.95 (t, *J* = 7.6 Hz, 1H, ArH), 3.62-3.52 (m, 1H, CH), 2.40 (s, 3H, CH_3_), 1.40 (d, *J* = 2.0 Hz, 3H, CH_3_), 1.14 (d, *J* = 6.6 Hz, 3H, CH_3_). ^13^C NMR (100 MHz, CDCl_3_) *δ* (ppm): 145.41, 142.24, 135.62, 134.97, 133.40, 129.41, 128.63, 127.24, 126.30, 124.94, 124.40, 58.01, 24.24, 21.15, 18.48.

**IH-IPT:** A colorless oily (1.00 g, 75%). ^1^H NMR (400 MHz, CDCl_3_) *δ* (ppm): 7.28 (s, 1H, ArH), 7.20-7.13 (m, 3H, ArH), 7.11-7.02 (m, 3H, ArH), 3.73-3.66 (m, 1H), 3.62-3.55 (m, 1H), 1.35-1.30 (m, 6H, CH_3_), 1.11 (s, 3H, CH_3_), 1.09 (d, *J* = 1.6 Hz, 3H, CH_3_). ^13^C NMR (100 MHz, CDCl_3_) *δ* (ppm): 146.34, 143.62, 142.61, 136.04, 134.10, 129.12, 127.21, 126.31, 125.07, 125.05, 124.74, 124.70, 58.40, 27.60, 25.06, 23.96, 22.21, 21.19.

**MM-MPT:** A white solid (1.50 g, 96%). mp: 86-88 ^o^C. ^1^H NMR (400 MHz, CDCl_3_) *δ* (ppm): 7.10 (d, *J* = 8.0 Hz, 4H, ArH), 7.04-6.94 (m, 2H, ArH), 3.12-2.99 (m, 3H, CH_3_), 2.45 (s, 6H, CH_3_). ^13^C NMR (100 MHz, CDCl_3_) *δ* (ppm): 145.58, 134.35, 133.36, 129.21, 124.92, 124.81, 40.42, 18.06. MS (EI) calcd. for C_15_H_15_NS [M+H]^+^: 241.09; Found: 241.05. Anal. calcd. for C_15_H_15_NS: C, 74.65; H, 6.26; N, 5.80. Found: C, 74.39; H, 6.27; N, 5.67.

**IM-MPT:** A white solid (1.00 g, 73%). mp: 42-44 ^o^C. ^1^H NMR (400 MHz, CDCl_3_) *δ* (ppm): 7.18-6.93 (m, 6H, ArH), 3.85-3.74 (m, 1H, CH), 3.01 (s, 3H, CH_3_), 2.46 (s, 3H, CH_3_), 1.35 (d, *J* = 7.0 Hz, 3H, CH_3_), 1.16 (d, *J* = 8.0 Hz, 3H, CH_3_). ^13^C NMR (100 MHz, CDCl_3_) *δ* (ppm): 145.75, 145.56, 143.92, 135.13, 133.87, 133.82, 129.04, 125.56, 125.07, 124.80, 124.74, 124.59, 41.59, 27.28, 25.04, 22.88, 17.75. MS (EI) calcd. for C_17_H_19_NS [M+H]^+^: 269.12; Found: 269.10. Anal. calcd. for C_17_H_19_NS: C, 75.79; H, 7.11; N, 5.20. Found: C, 75.59; H, 7.26; N, 5.12.

**II-MPT:** A white solid (1.00 g, 89%). mp: 126-128 ^o^C. ^1^H NMR (400 MHz, CDCl_3_) *δ* (ppm): 7.18-7.06 (m, 6H, ArH), 3.86-3.79 (m, 2H, CH), 3.00 (s, 3H, CH_3_), 1.35 (d, *J* = 8.0 Hz, 6H, CH_3_), 1.16 (d, *J* = 8.0 Hz, 6H, CH_3_). ^13^C NMR (100 MHz, CDCl_3_) *δ* (ppm): 146.21, 143.97, 134.12, 125.66, 124.74, 124.56, 77.22, 42.85, 27.21, 24.93, 23.18. MS (EI) calcd. for C_19_H_23_NS [M+H]^+^: 297.15; Found: 297.15. Anal. calcd. for C_19_H_23_NS: C, 76.72; H, 7.79; N, 4.71. Found: C, 76.57; H, 7.95; N, 4.72.

**MM-IPT:** A white solid (1.00 g, 86%). mp: 72-74 ^o^C. ^1^H NMR (400 MHz, CDCl_3_) *δ* (ppm): 7.17 (d, *J* = 8.0 Hz, 2H, ArH), 7.07 (d, *J* = 8.0 Hz, 2H, ArH), 6.96 (t, *J* = 7.6 Hz, 2H, ArH), 3.83-3.77 (m, 1H, CH), 2.49 (s, 6H, CH_3_), 0.97 (d, *J* = 8.0 Hz, 6H, CH_3_). ^13^C NMR (100 MHz, CDCl_3_) *δ* (ppm): 144.40, 137.83, 136.53, 128.89, 125.42, 124.95, 52.55, 22.26, 18.91. MS (EI) calcd. for C_17_H_19_NS [M+H]^+^: 269.12; Found: 269.05. Anal. calcd. for C_17_H_19_NS: C, 75.79; H, 7.11; N, 5.20. Found: C, 75.70; H, 7.19; N, 5.22.

**IM-IPT:** A white solid (1.00 g, 89%). mp: 62-64 ^o^C. ^1^H NMR (400 MHz, CDCl_3_) *δ* (ppm): 7.22-7.04 (m, 6H, ArH), 3.85-3.74 (m, 2H, CH), 3.00 (s, 3H, CH_3_), 1.35 (d, *J* = 8.0 Hz, 6H, CH_3_), 1.16 (d, *J* = 8.0 Hz, 6H, CH_3_). ^13^C NMR (100 MHz, CDCl_3_) *δ* (ppm): 146.21, 143.97, 134.12, 125.66, 124.74, 124.56, 77.22, 42.85, 27.21, 24.93, 23.18. MS (EI) calcd. for C_19_H_23_NS [M+H]^+^: 297.15; Found: 297.10. Anal. calcd. for C_19_H_23_NS: C, 76.72; H, 7.79; N, 4.71. Found: C, 76.65; H, 7.87; N, 4.65.

**II-IPT:** A white solid (1.20 g, 80%). mp: 84-86 ^o^C. ^1^H NMR (400 MHz, CDCl_3_) *δ* (ppm): 7.21-7.18 (m, 4H, ArH), 7.09 (t, *J* = 7.6 Hz, 2H, ArH), 4.13-4.06 (m, 2H, CH), 3.79-3.72 (m, 1H, CH), 1.35 (d, *J* = 6.9 Hz, 6H, CH_3_), 1.07 (d, *J* = 7.0 Hz, 6H, CH_3_), 0.94 (d, *J* = 6.4 Hz, 6H, CH_3_). ^13^C NMR (100 MHz, CDCl_3_) *δ* (ppm): 148.97, 142.37, 136.03, 125.47, 124.91, 124.38, 77.21, 51.39, 26.97, 25.89, 22.85, 22.63. MS (EI) calcd. for C_21_H_27_N [M+H]^+^: 325.18; Found: 325.10. Anal. calcd. for C_21_H_27_N: C, 77.49; H, 8.36; N, 4.30. Found: C, 77.42; H, 8.30; N, 4.36.

**NMR spectra and HPLC data of compounds**

**Figure S1** ^1^H NMR spectrum of II-DPA.

**Figure S2** ^13^C NMR spectrum of II-DPA.

**Figure S3** ^1^H NMR spectrum of MH-HPT.

# **Figure S4** ^13^C NMR spectrum of MH-HPT.

# **Figure S5** ^1^H NMR spectrum of IH-HPT.

# **Figure S6** ^13^C NMR spectrum of IH-HPT.

**Figure S7** ^1^H NMR spectrum of HH-MPT.

**Figure S8** ^13^C NMR spectrum of HH-MPT.

**Figure S9** ^1^H NMR spectrum of HH-IPT.

**Figure S10** ^13^C NMR spectrum of HH-IPT.

**Figure S11** ^1^H NMR spectrum of MM-HPT.

**Figure S12** ^13^C NMR spectrum of MM-HPT.

**Figure S13**^1^H NMR spectrum of IM-HPT.

# **Figure S14** ^13^C NMR spectrum of IM-HPT.

**Figure S15** ^1^H NMR spectrum of II-HPT.

**Figure S16** ^13^C NMR spectrum of II-HPT.

**Figure S17** ^1^H NMR spectrum of MH-MPT.

**Figure S18** ^13^C NMR spectrum of MH-MPT.

# **Figure S19** ^1^H NMR spectrum of IH-MPT.

**Figure S20** ^13^C NMR spectrum of IH-MPT.

**Figure S21** ^1^H NMR spectrum of MH-IPT.

# **Figure S22** ^13^C NMR spectrum of MH-IPT.

**Figure S23** ^1^H NMR spectrum of IH-IPT.

# **Figure S24** ^13^C NMR spectrum of IH-IPT.

**Figure S25** ^1^H NMR spectrum of MM-MPT.

# **Figure S26** ^13^C NMR spectrum of MM-MPT.

**Figure S27** ^1^H NMR spectrum of IM-MPT.

# **Figure S28** ^13^C NMR spectrum of IM-MPT.

**Figure S29** ^1^H NMR spectrum of II-MPT.

# **Figure S30** ^13^C NMR spectrum of II-MPT.

**Figure S31** ^1^H NMR spectrum of MM-IPT.

# **Figure S32**^13^C NMR spectrum of MM-IPT.

**Figure S33** ^1^H NMR spectrum of IM-IPT.

# **Figure S34** ^13^C NMR spectrum of IM-IPT.

**Figure S35** ^1^H NMR spectrum of II-IPT.

**Figure S36** ^13^C NMR spectrum of II-IPT.

**Figure S37** HPLC curve of HH-HPT.


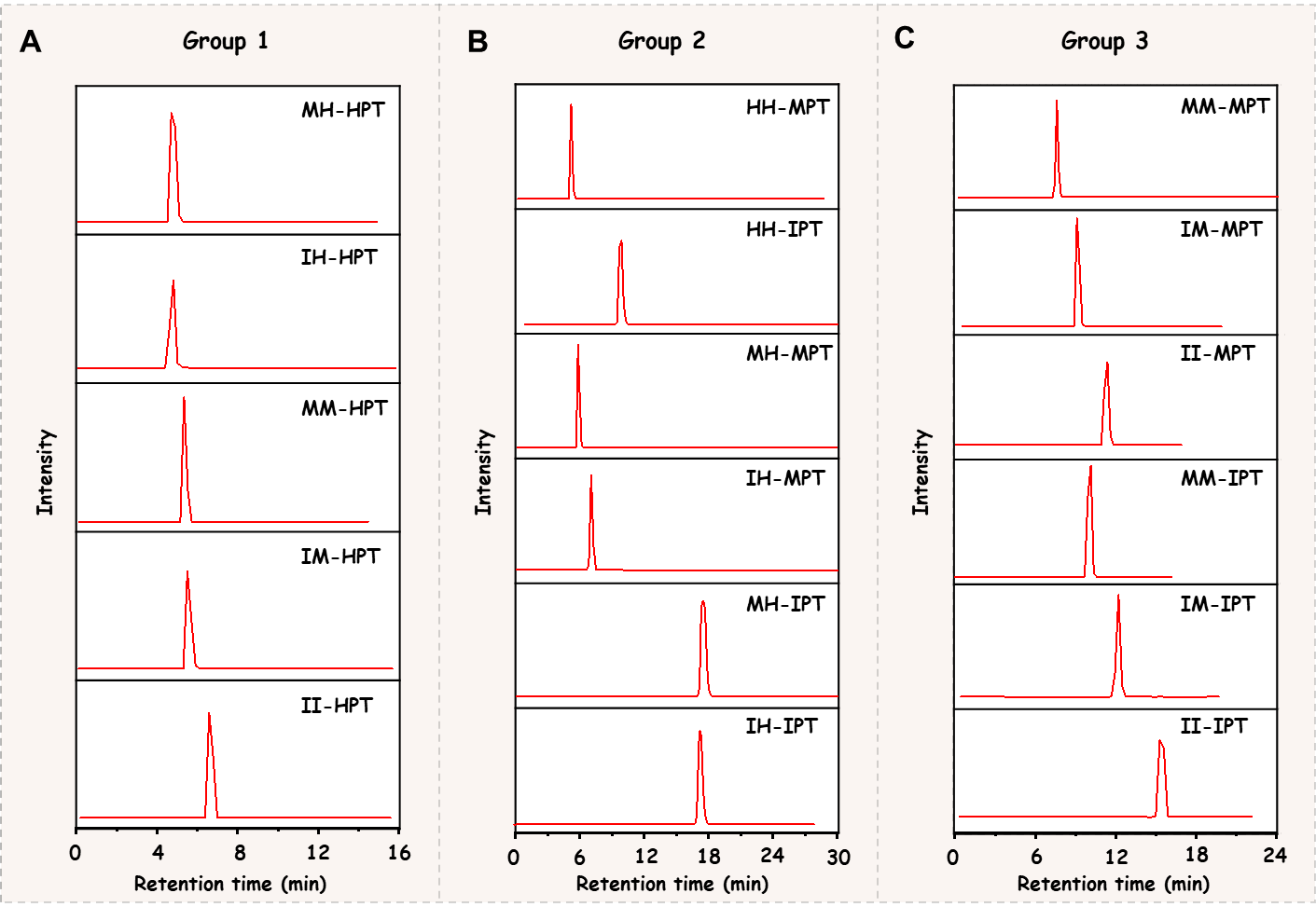


**Figure S38** HPLC curve of phenothiazine derivatives in (A) Group 1, (B) Group 2 and (C) Group 3.

**Table S1 Crystal data of phenothiazine derivatives in Group 1.**

| **Compound** | **MH-HPT** | **IH-HPT** | **IM-HPT** |
| --- | --- | --- | --- |
| **Formula** | C_13_H_11_NS | C_15_H_15_NS | C_16_H_17_NS |
| **Wavelength (Å)** | 0.71073 | 0.71073 | 0.71073 |
| **Space Group** | P 21/c | P 21/c | P 21 21 21 |
| **Cell Lengths (Å)** | a = 9.0752 (3)  b = 8.9707 (3)  c = 13.4991 (4) | a = 19.968 (3)  b = 7.7334 (11)  c = 8.3685 (12) | a = 8.1235 (3)  b = 8.4076 (3)  c = 19.8254 (7) |
| **Cell Angles (°)** | α = 90  β = 102.298 (3)  γ = 90° | α = 90  β = 90  γ = 90 | α = 90  β = 90  γ = 90 |
| **Cell Volume (Å^3^)** | 1073.76 (6) | 1292.3 (3) | 1354.06 (8) |
| **Z** | 4 | 4 | 4 |
| **Density (g/cm^3^)** | 1.319 | 1.240 | 1.253 |
| **F (000)** | 448.0 | 512 | 544.0 |
| **h_max_, k_max_, I_max_.** | 11, 11, 16 | 24, 9, 10 | 10, 10, 24 |
| **T_min_, T_max_** | 0.641, 1.000 | 0.588, 0.746 | 0.511, 1.000 |
| **R_1_** | 0.04 | 0.04 | 0.05 |
| **wR_2_** | 0.11 | 0.10 | 0.12 |
| **GOOF** | 1.08 | 1.08 | 1.10 |
| **CCDC** | 2349605 | 2349606 | 2349609 |

**Table S2 Crystal data of phenothiazine derivatives in Group 2.**

| **Name** | **HH-MPT** | **MH-MPT** | **IH-MPT** |
| --- | --- | --- | --- |
| **Formula** | C_13_H_11_NS | C_14_H_13_NS | C_16_H_17_NS |
| **Wavelength (Å)** | 0.71073 | 0.71073 | 0.71073 |
| **Space Group** | Cmc21 | Pna21 | P1 21/c 1 |
| **Cell Lengths (Å)** | a = 11.317 (2)  b = 14.653 (3)  c = 6.6724 (13) | a = 15.5782 (10)  b = 11.1023 (8)  c = 6.9354 (4) | a = 12.2465 (10)  b = 7.9861 (6)  c = 14.8962 (12) |
| **Cell Angles (°)** | α = 90  β = 90  γ = 90 | α = 90  β = 90  γ = 90 | α = 90  β = 111.330 (9)  γ = 90 |
| **Cell Volume (Å^3^)** | 1106.5 (4) | 1199.50 (13) | 1357.1 (2) |
| **Z** | 8 | 4 | 4 |
| **Density (g/cm^3^)** | 1.280 | 1.259 | 1.250 |
| **F (000)** | 488.6 | 480.0 | 544.7 |
| **h_max_, k_max_, I_max_.** | 13, 17, 7 | 21, 15, 9 | 14, 9 17 |
| **T_min_, T_max_** | 0.618, 0.746 | 0.885, 1.000 | 0.917, 1.000 |
| **R_1_** | 0.03 | 0.03 | 0.04 |
| **wR_2_** | 0.09 | 0.09 | 0.12 |
| **GOOF** | 1.02 | 1.07 | 1.03 |
| **CCDC** | 2349607 | 2349610 | 2349611 |

**Table S3.** Crystal data of phenothiazine derivatives in Group 3.

| **Name** | **MM-MPT** | **IM-MPT** | **II-MPT** | **MM-IPT** | **IM-IPT** |
| --- | --- | --- | --- | --- | --- |
| **Formula** | C_15_H_15_NS | C_17_H_19_NS | C_19_H_23_NS | C_17_H_19_NS | C_19_H_23_NS |
| **Wavelength (Å)** | 0.71073 | 0.71073 | 0.71073 | 0.71073 | 0.71073 |
| **Space Group** | P b c a | P 21 21 21 | P-1 | P 1 21/c 1 | P -1 |
| **Cell Lengths (Å)** | a = 8.0527 (6)  b = 14.7485 (11)  c = 21.1880 (14) | a = 7.1388 (7)  b = 9.3893 (7)  c = 22.359 (3) | a = 9.134 (3)  b = 9.601 (3)  c =1 0.298 (3) | a = 11.694 (2)  b = 8.2136 (15)  c = 15.796 (3) | a = 9.3072 (15)  b = 9.6424 (16)  c = 10.5012 (17) |
| **Cell Angles (°)** | α = 90  β = 90  γ = 90 | α = 90  β = 90  γ = 90 | α = 82.095 (4)  β = 72.360 (4)  γ = 81.458 (4) | α = 90  β = 99.276 (3)  γ = 90 | α = 95.376 (2)  β = 110.853 (2)  γ = 100.335 (2) |
| **Cell Volume (Å^3^)** | 2516.4 (3) | 1498.7 (3) | 847.0 (4) | 1497.4 (5) | 853.6 (2) |
| **Z** | 8 | 4 | 2 | 4 | 2 |
| **Density (g/cm^3^)** | 1.274 | 1.194 | 1.166 | 1.195 | 1.157 |
| **F (000)** | 0.233 | 576.0 | 320.3 | 576.7 | 320.3 |
| **h_max_, k_max_, I_max_.** | 10,20,29 | 9,12,30 | 10,11,12 | 13,9,18 | 11,11,12 |
| **T_min_, T_max_** | 0.950, 1.000 | 0.686, 1.000 | 0.624, 0.746 | 0.654, 0.746 | 0.982, 0.982 |
| **R_1_** | 0.05 | 0.05 | 0.04 | 0.05 | 0.05 |
| **wR_2_** | 0.14 | 0.11 | 0.14 | 0.20 | 0.17 |
| **GOOF** | 1.02 | 1.03 | 1.07 | 1.10 | 1.06 |
| **CCDC** | 2349612 | 2349613 | 2349614 | 2349615 | 2349616 |

**Supplementary data**


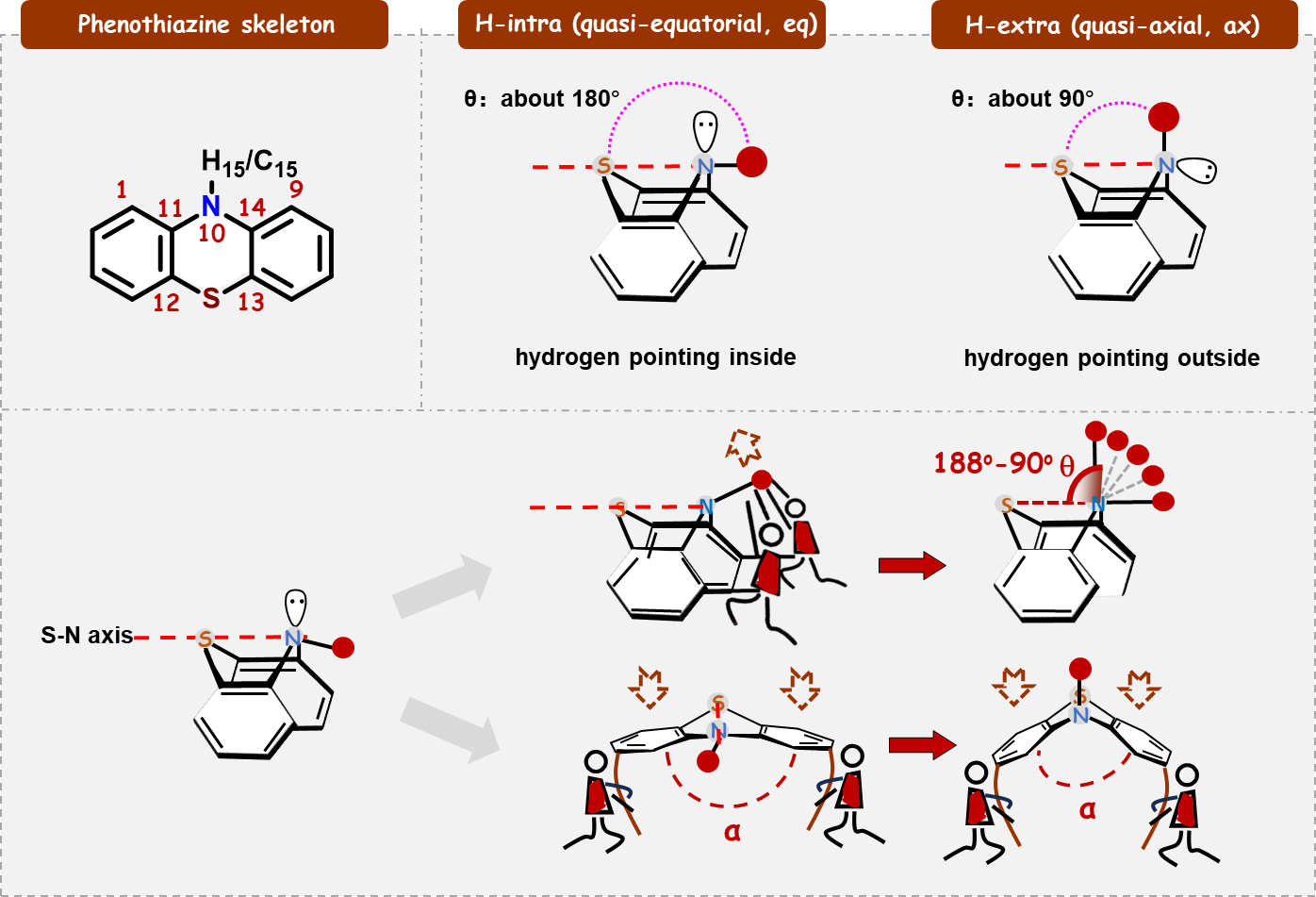


**Figure S39.** Two conformations of phenothiazine derivatives: H-intra (quasi-equatorial, *eq*) and H-extra (quasi-axial, *ax*), and the adjustment of folded angles (θ) by steric effect.


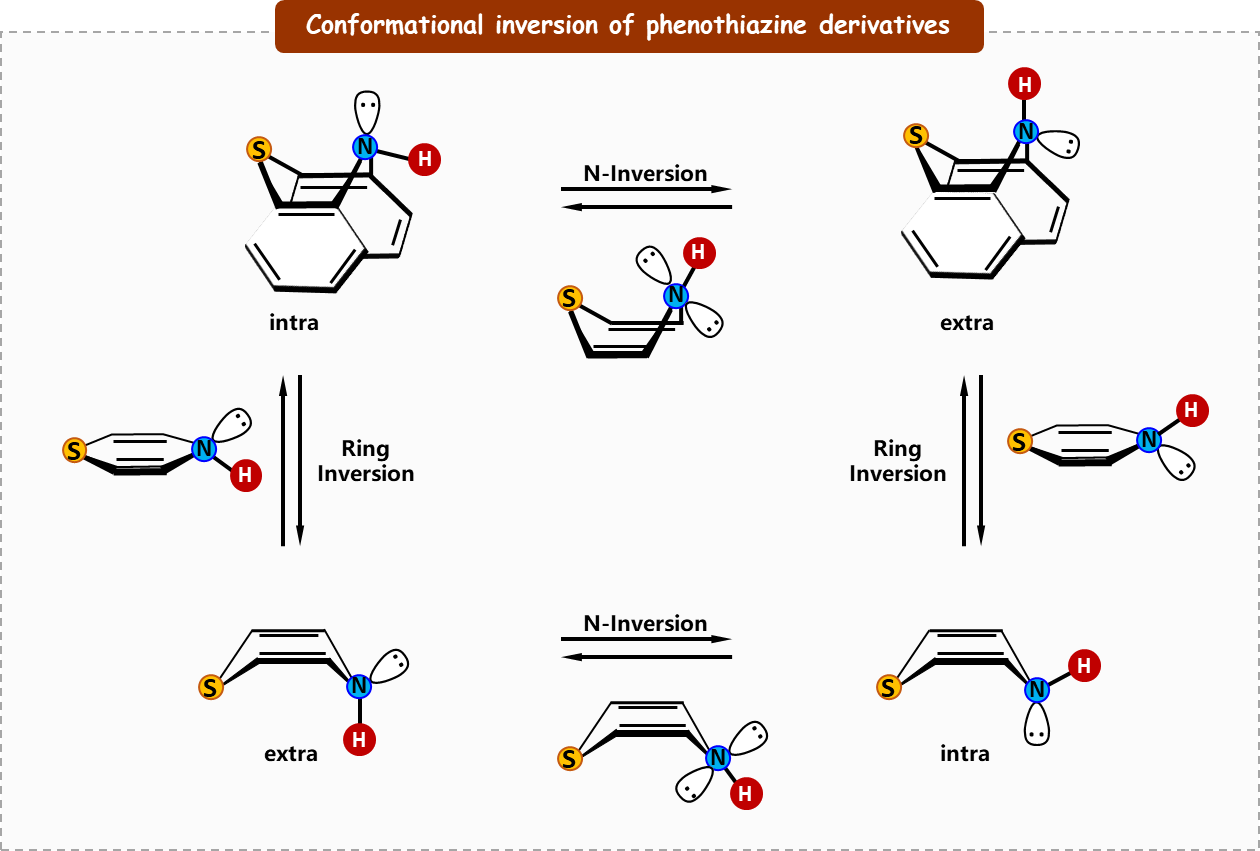


**Figure S40.** Conformational inversion processes of phenothiazine moiety.


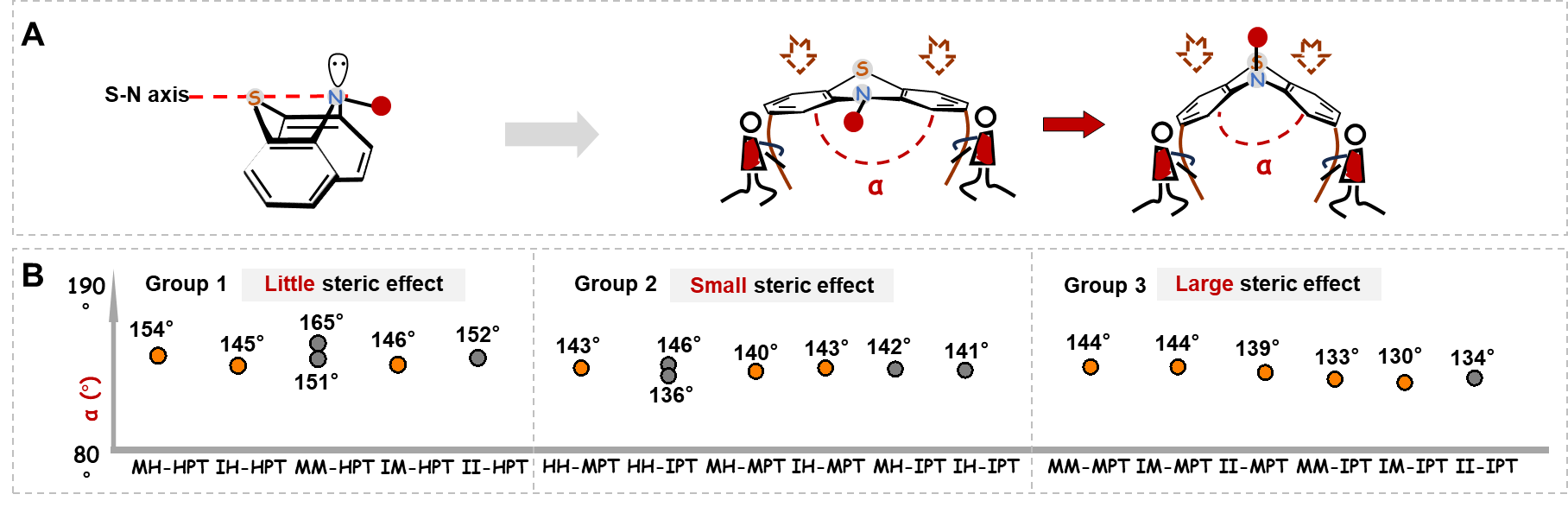


**Figure S41.** (A) The adjustment of dihedral angle α by steric effect. (B) Dependence relationship between steric hindrance and dihedral angle α for phenothiazine derivatives in Group1-3.


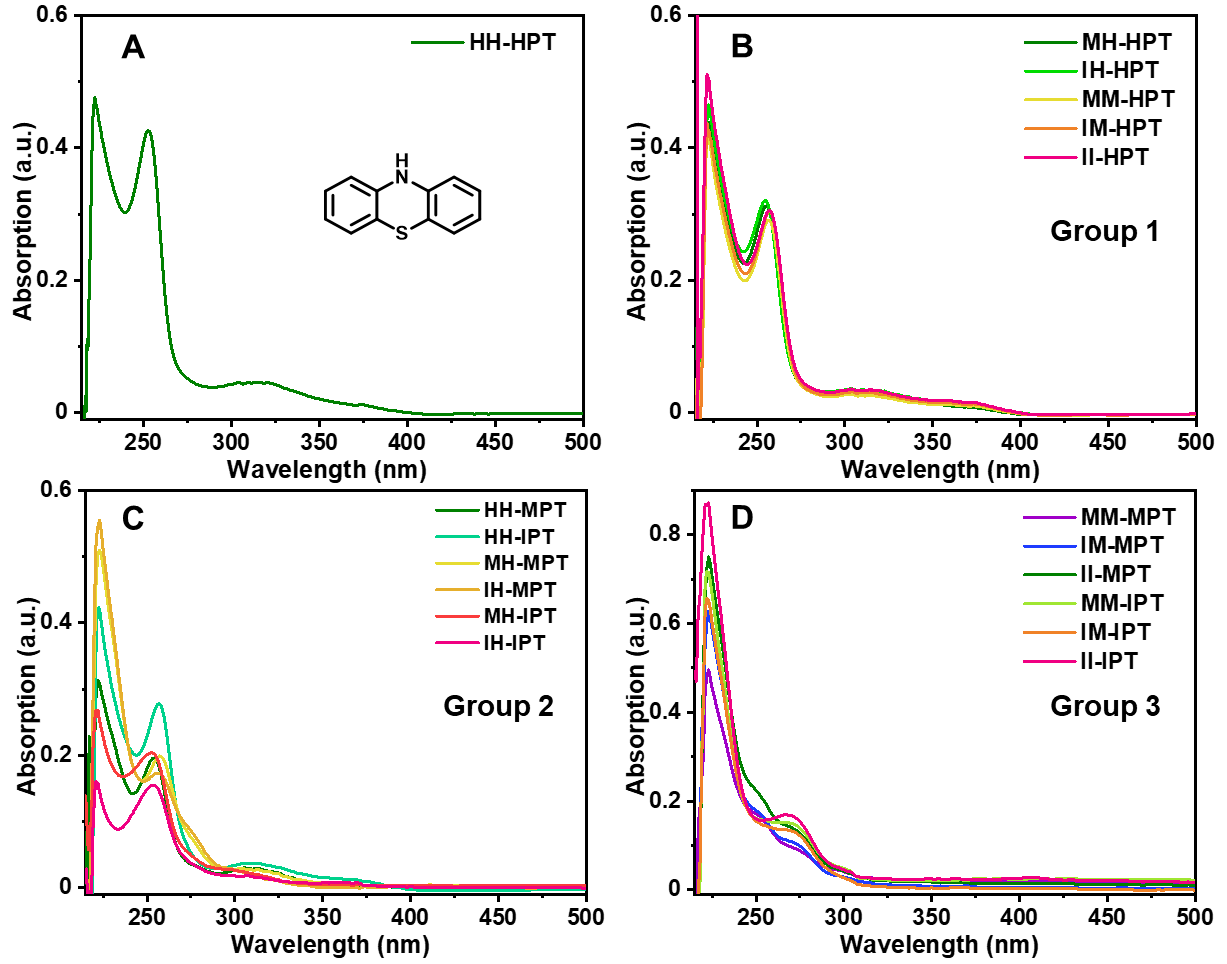


**Figure S42.** UV-Vis absorption spectra of (A) HH-HPT, and phenothiazine derivatives in (B) Group 1, (C) Group 2, and (D) Group 3 in DCM solution (10 *μ*M).


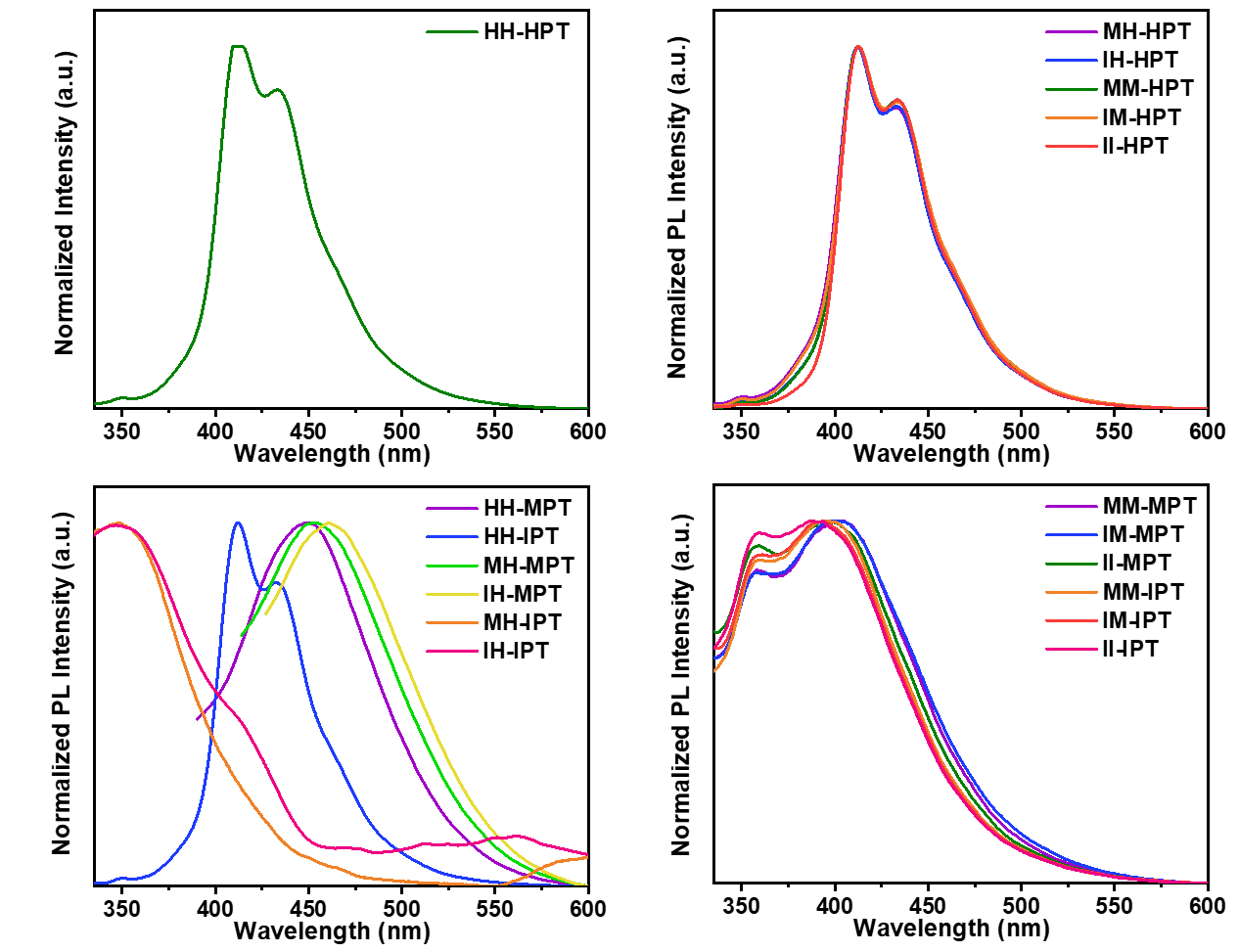


**Figure S43.** Photoluminescence spectra of (A) HH-HPT, and phenothiazine derivatives in (B) Group 1, (C) Group 2, and (D) Group 3 in DCM solution (10 *μ*M) under ambient conditions (λ_ex_: 315 nm).


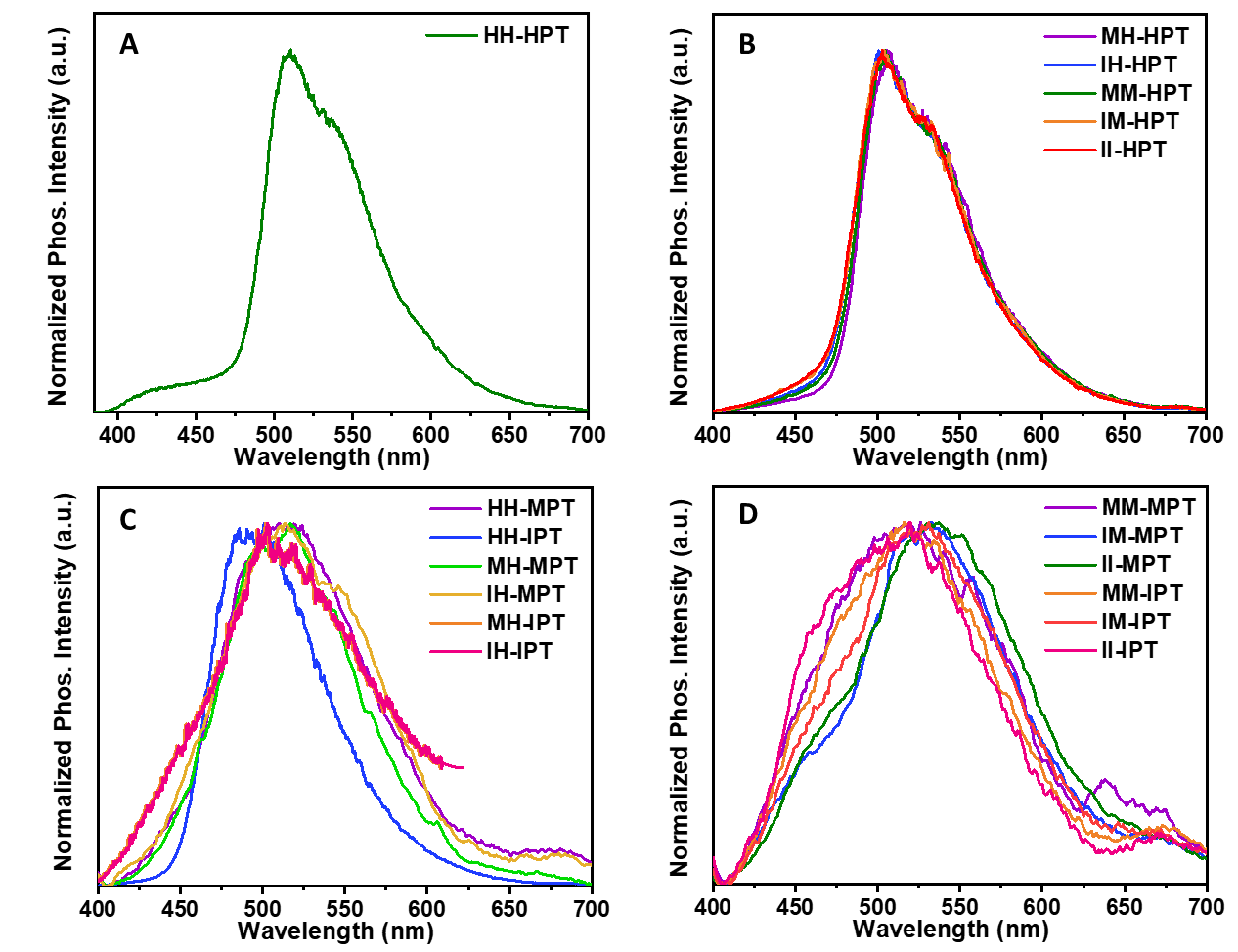


**Figure S44.** Normalized phosphorescent spectra of (A) HH-HPT, and phenothiazine derivatives in (B) Group 1, (C) Group 2, and (D) Group 3 in DCM solution (10 *μ*M) at 77 K (λ_ex_: 365 nm).


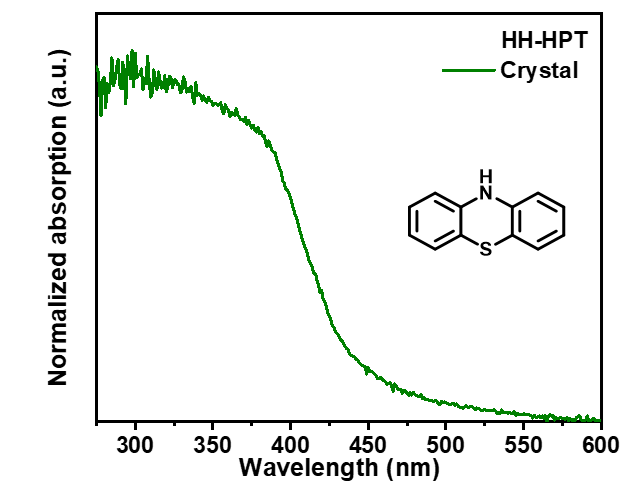


**Figure S45.** UV-Vis absorption spectra of HH-HPT crystal at room temperature.


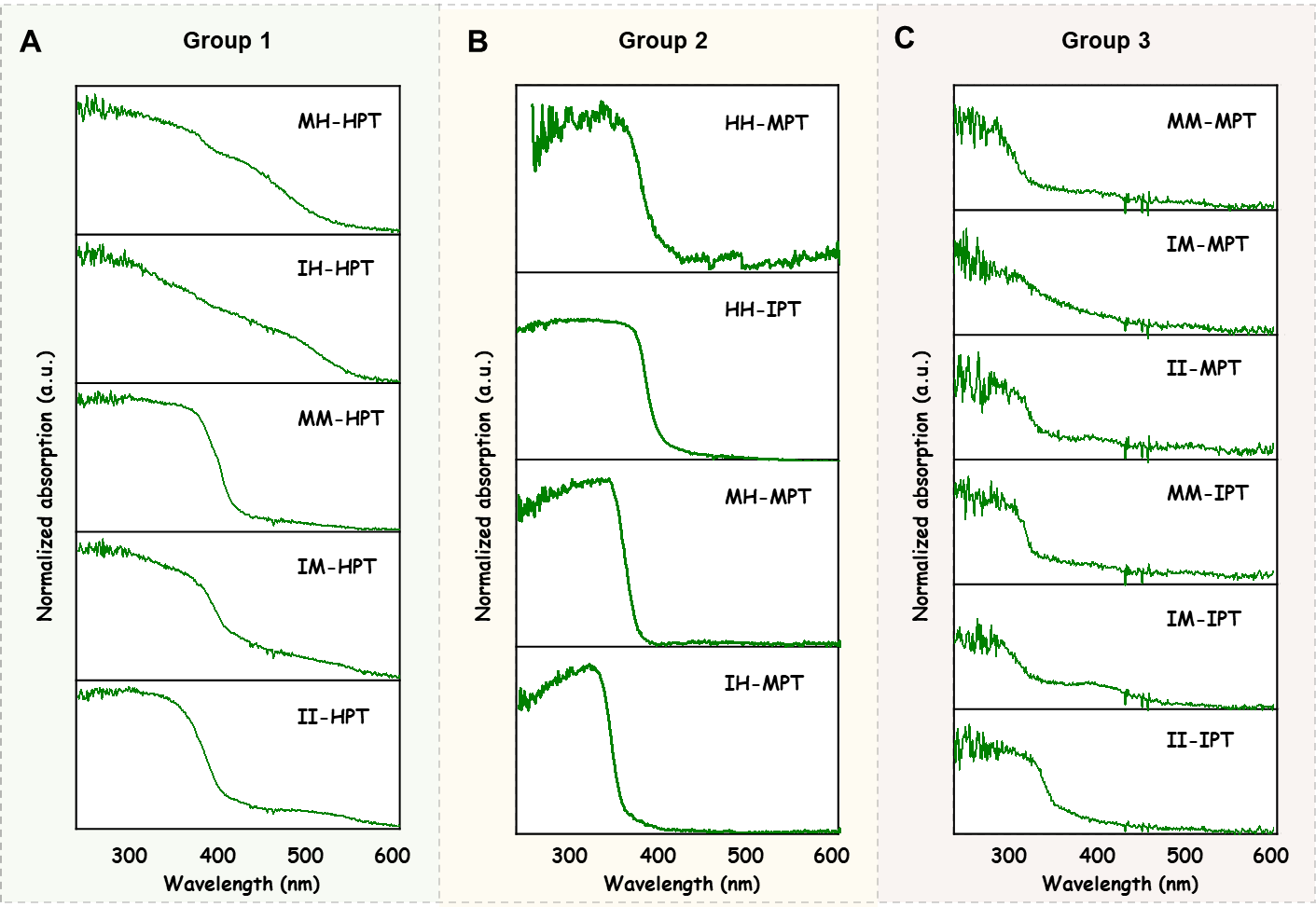


**Figure S46.** UV-Vis absorption spectra of phenothiazine derivatives in (A) Group 1, (B) Group 2, and (C) Group 3 at crystal state at room temperature.


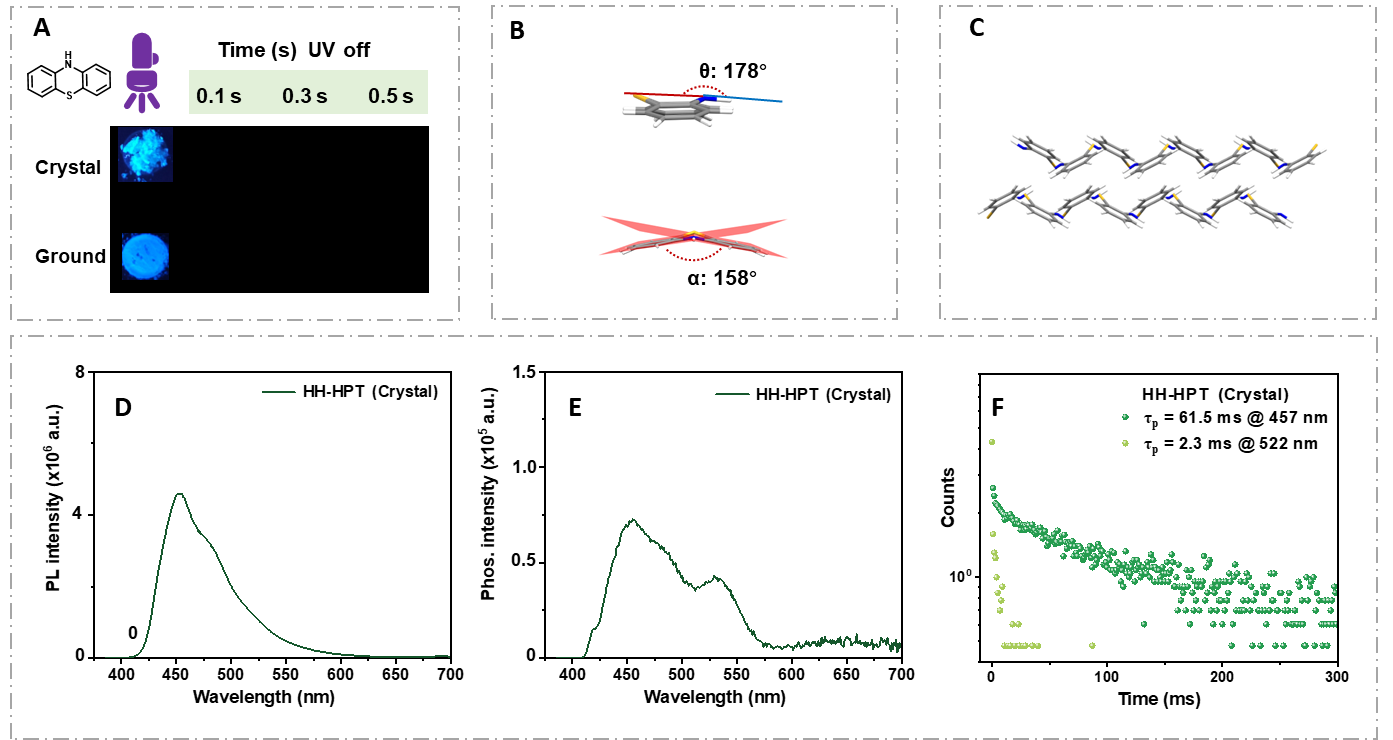


**Figure S47.** (A) Photographs of phenothiazine (HH-HPT) in crystal (top) and ground states (down) under different conditions. (B) The folding angle θ and dihedral angle α of HH-HPT, with molecular conformation extracted from single crystal. (C) Molecular packing of HH-HPT in single crystal. The steady-state PL spectra (D), phosphorescence spectra (E), and phosphorescence decay (F) of HH-HPT crystal (λ_ex,PL_: 365 nm, λ_ex,phos_.: 365 nm) at room temperature.


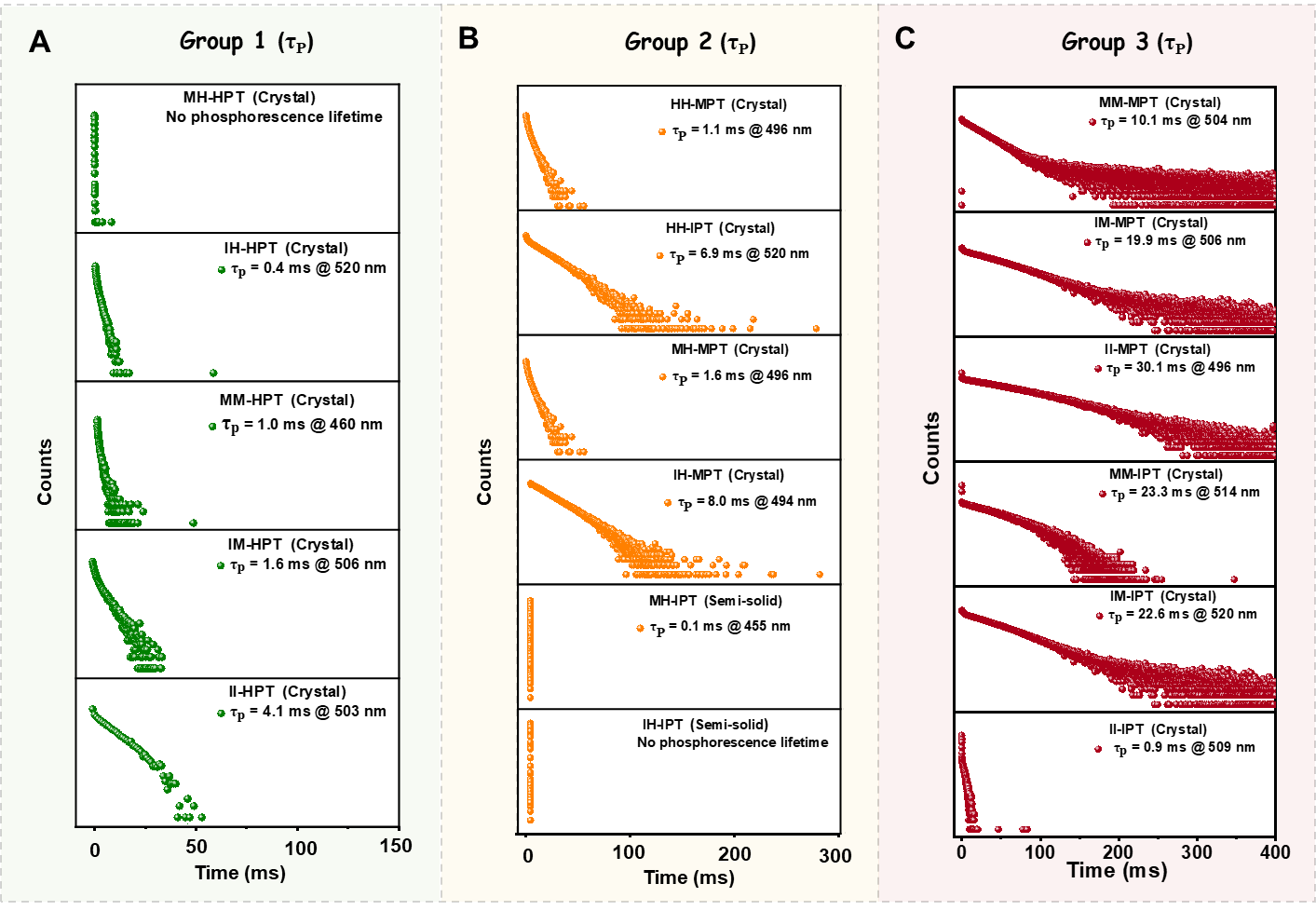


**Figure S48.** Phosphorescence decays of phenothiazine derivatives in (A) Group 1, (B) Group 2, and (C) Group 3 at crystal state (MH-IPT and IH-IPT with semi-solid states) at room temperature.


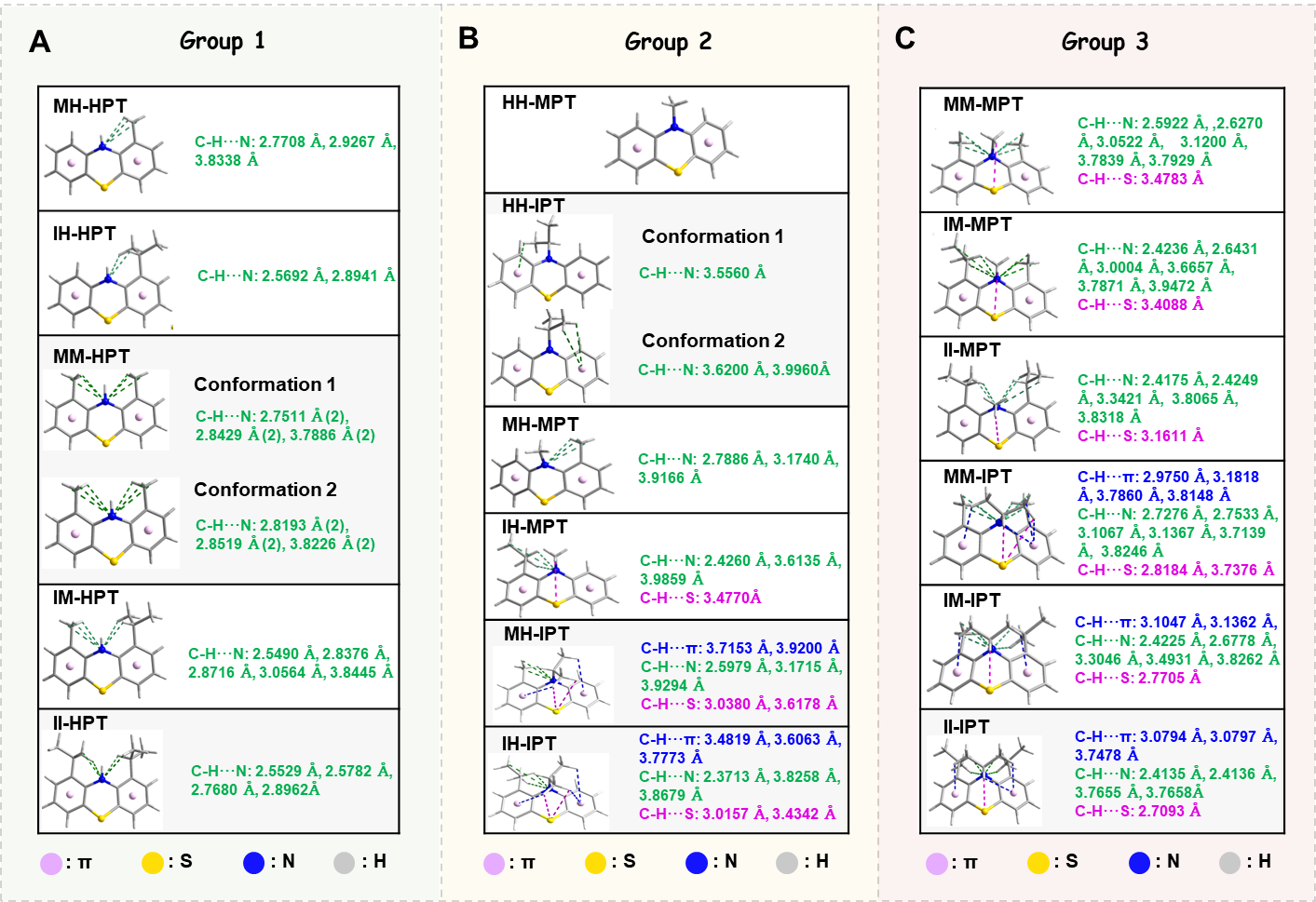


**Figure S49.** Intramolecular interactions of phenothiazine derivatives in (A) Group 1, (B) Group 2, and (C) Group 3 at crystal state.


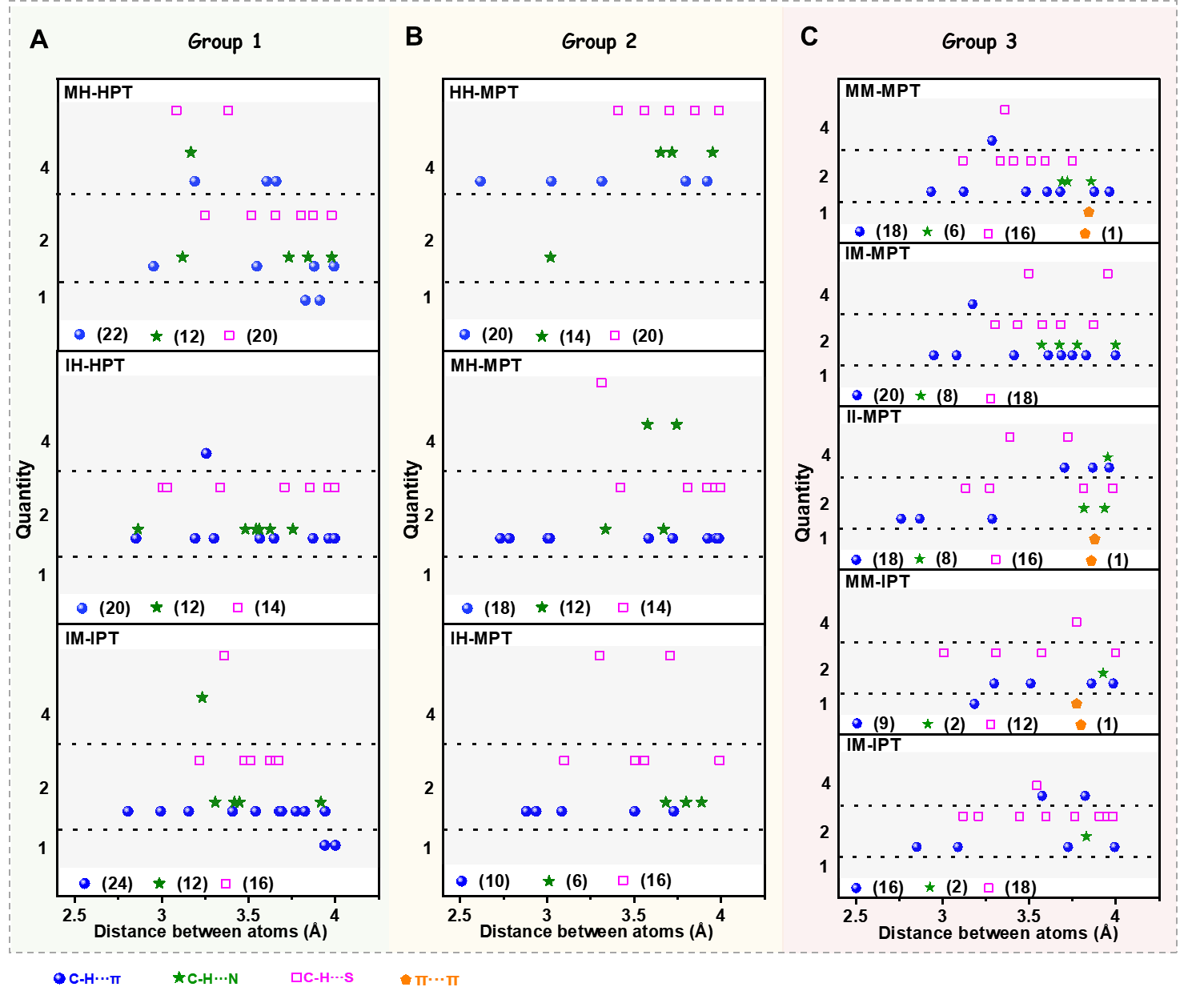


**Figure S50.** The intermolecular interactions of phenothiazine derivatives in (A) Group 1, (B) Group 2, and (C) Group 3 at crystal state (3×3×3 lattice).


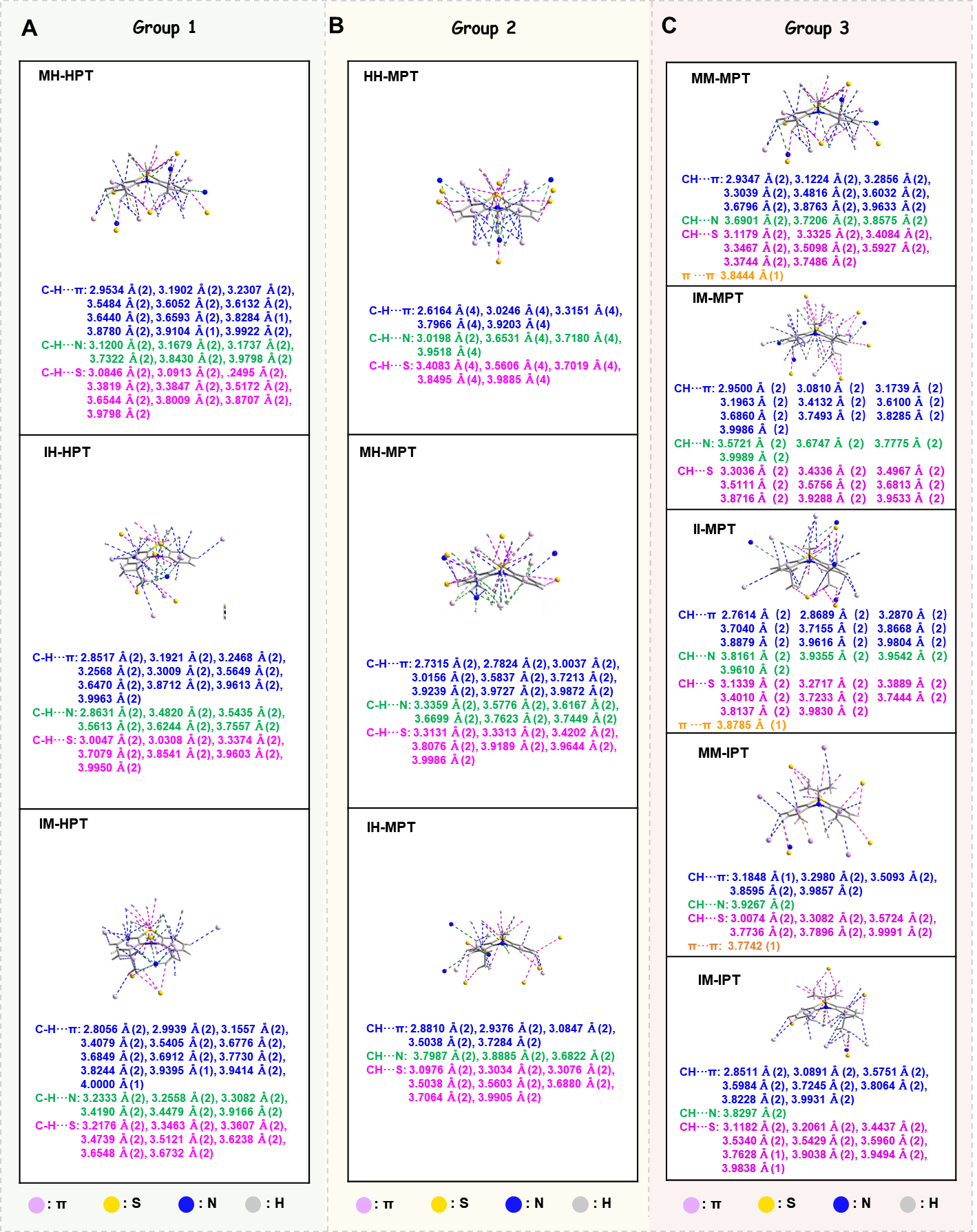


**Figure S51.** Intermolecular interactions diagram of phenothiazine derivatives in (A) Group 1, (B) Group 2, and (C) Group 3 at crystal state.


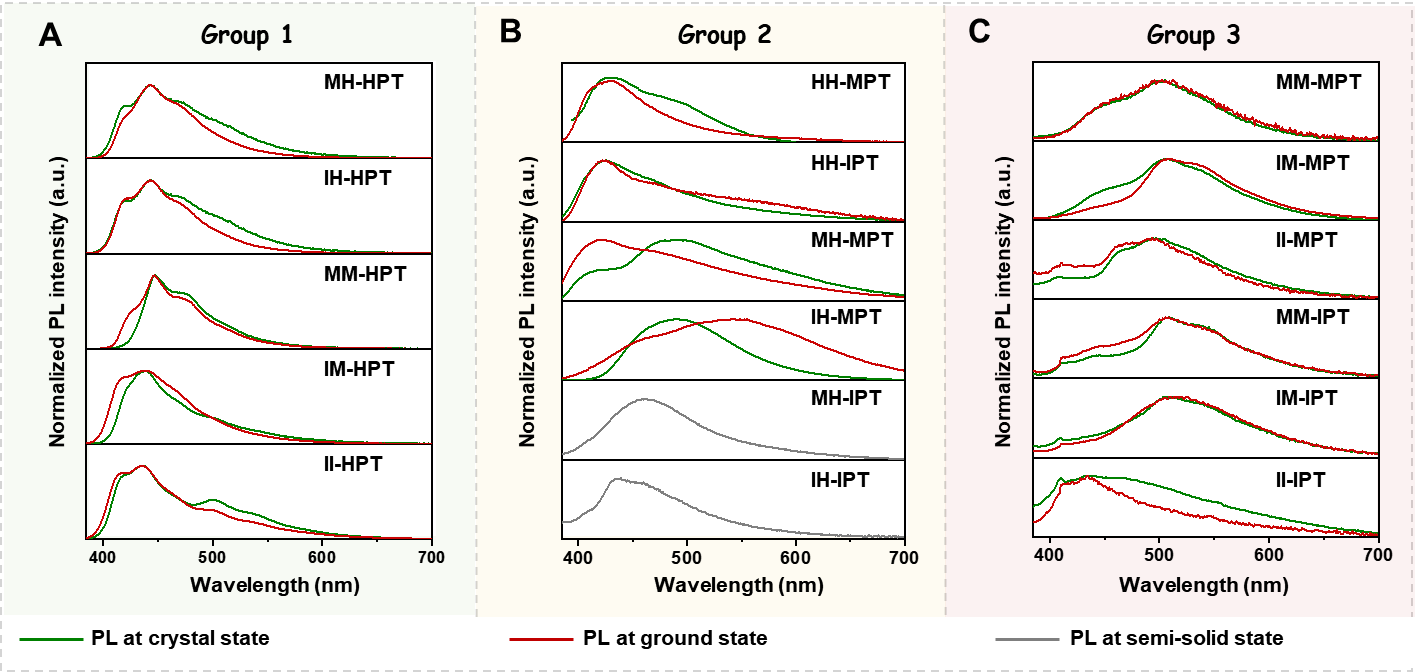


**Figure S52.** The normalized steady-state PL spectra of phenothiazine derivatives in Group 1 (A), Group 2 (B), and Group 3 (C) at crystal state at room temperature.


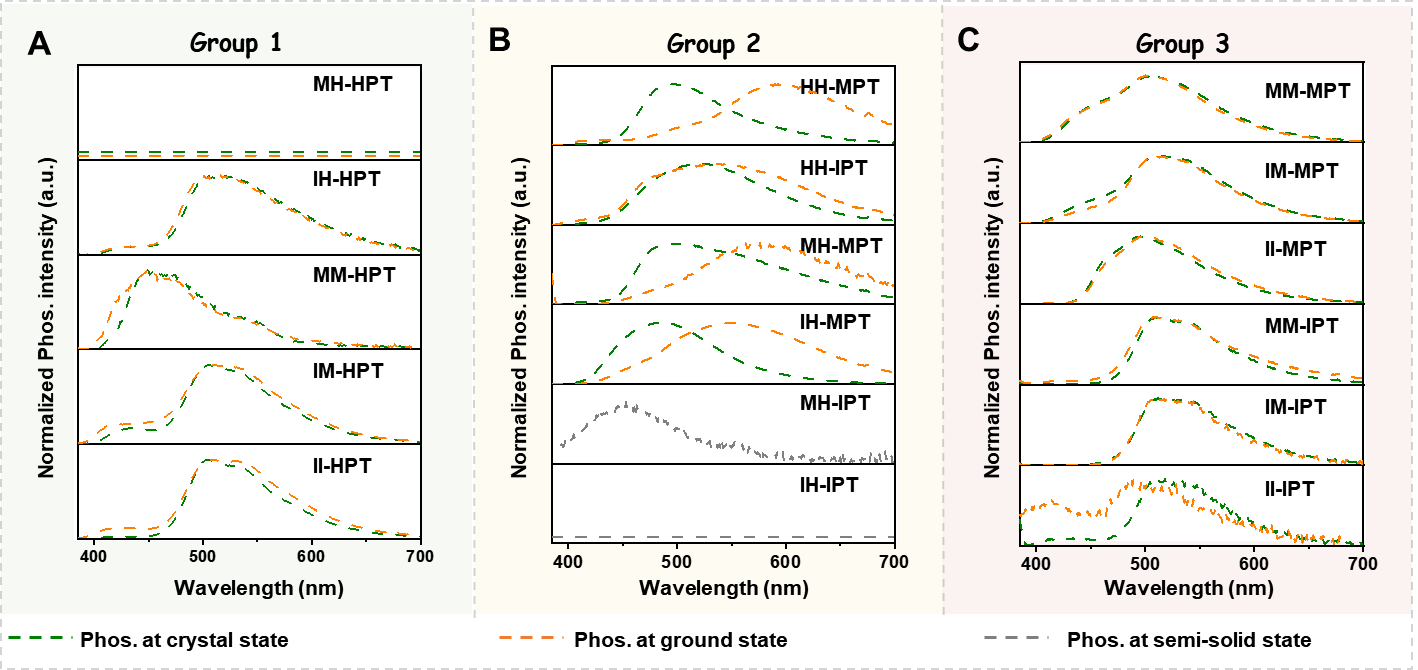


**Figure S53.** The normalized phosphorescence (Phos.) spectra of phenothiazine derivatives in Group 1 (A), Group 2 (B), and Group 3 (C) at ground states at room temperature.


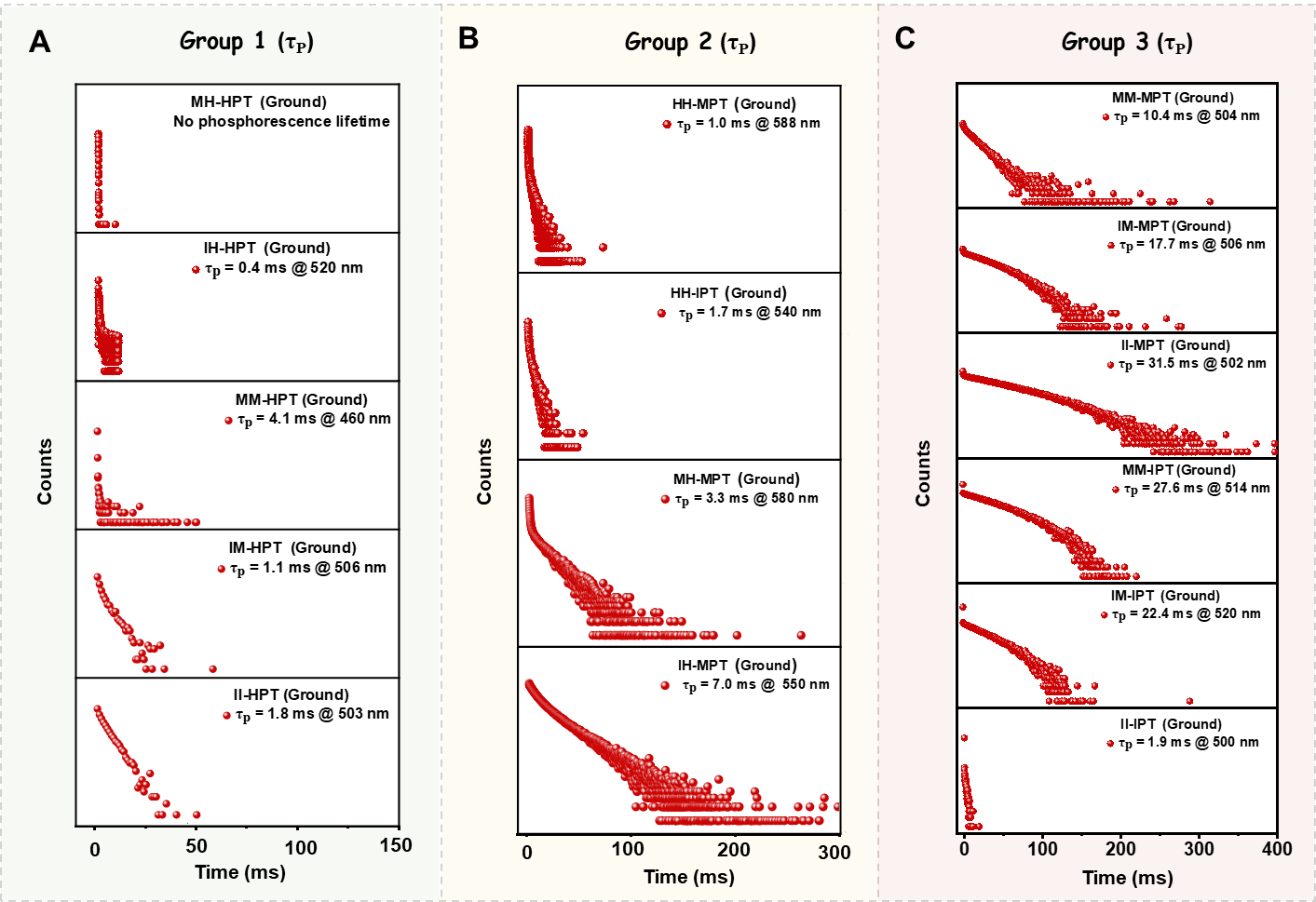


**Figure S54.** Phosphorescence decays of phenothiazine derivatives in (A) Group 1, (B) Group 2, and (C) Group 3 at ground state at room temperature.


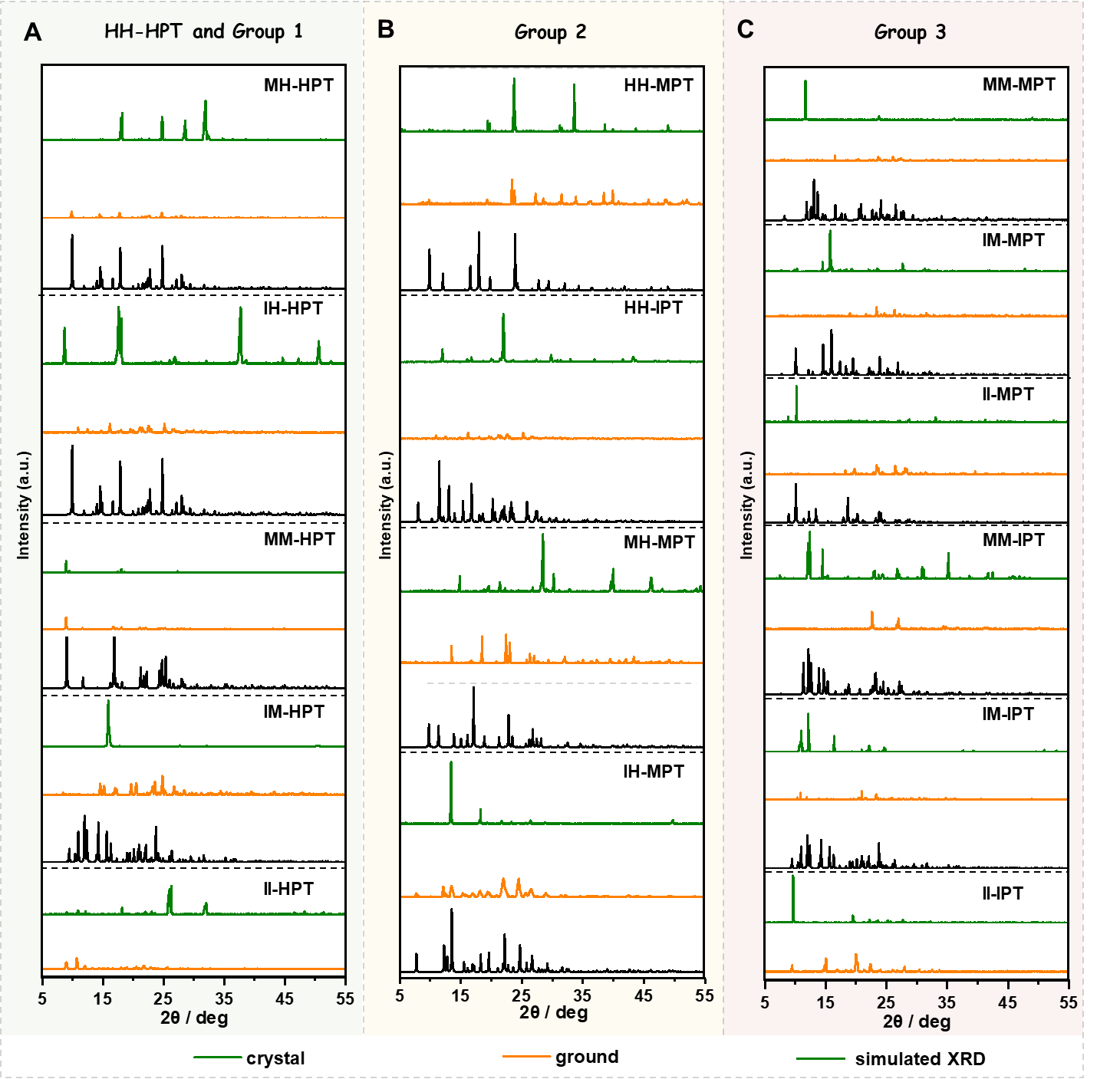


**Figure S55.** Powder X-ray diffraction (PXRD) patterns of phenothiazine derivatives in Group 1 and Group 3 (the simulated XRD pattern calculated from single-crystal X-ray data with Mercury 2022.2.0) at crystal state and ground state.


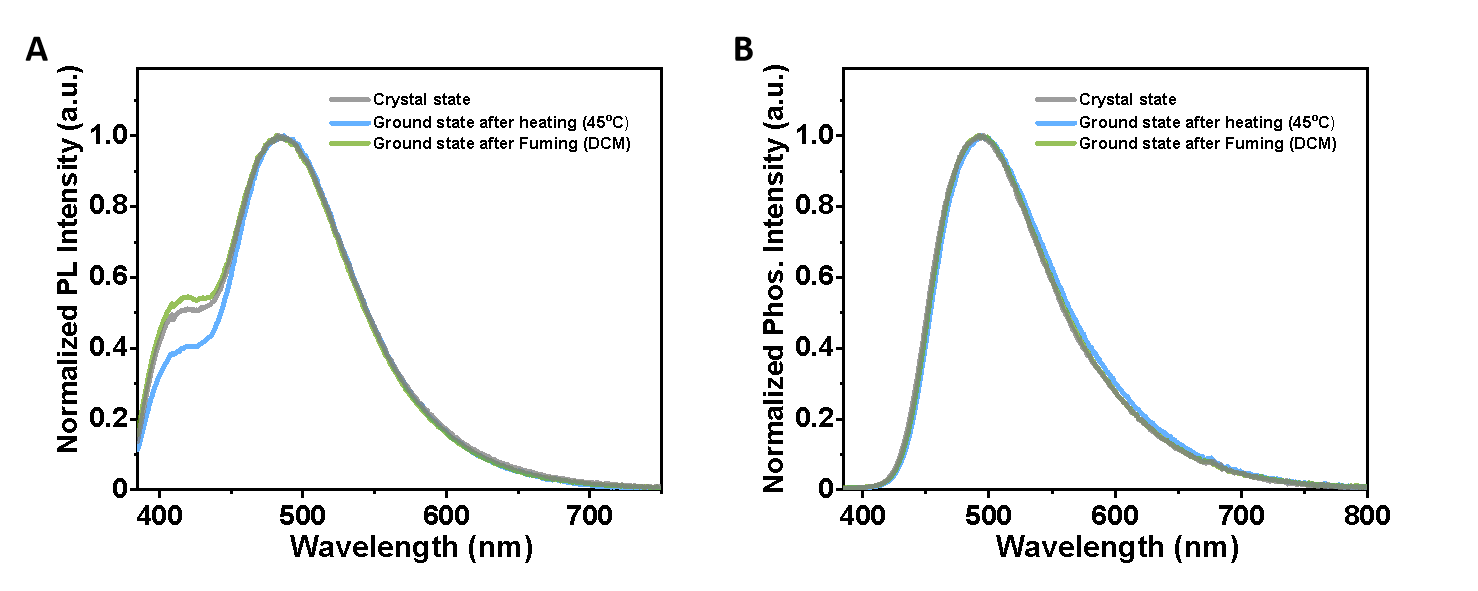


**Figure S56.** Normalized steady-state photoluminescence spectra (A) and phosphorescence spectra (B) of MH-MPT under different conditions (Excitation wavelength: 365 nm).


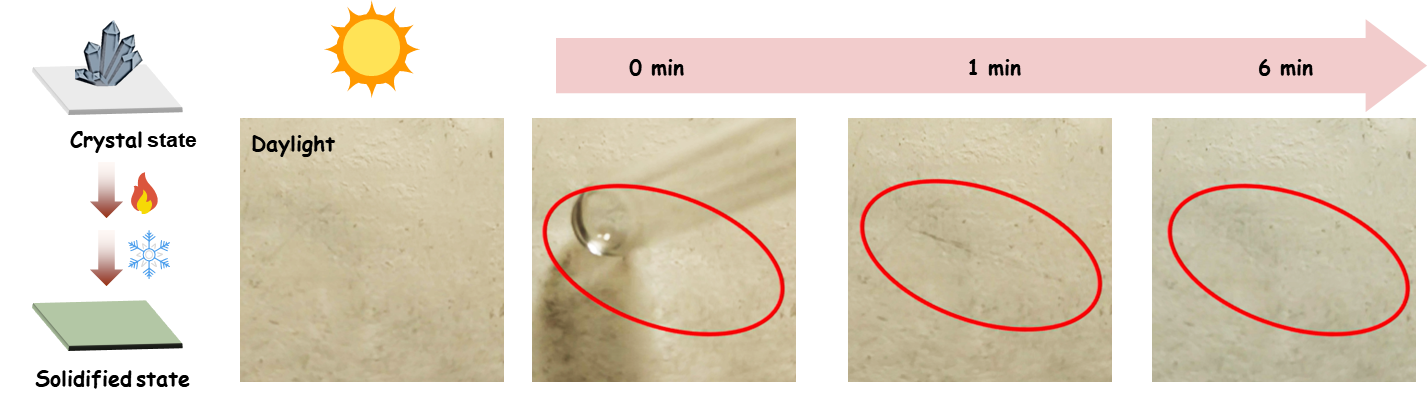


**Figure S57.** Photographs of IH-MPT film before and after force-stimulation under daylight.


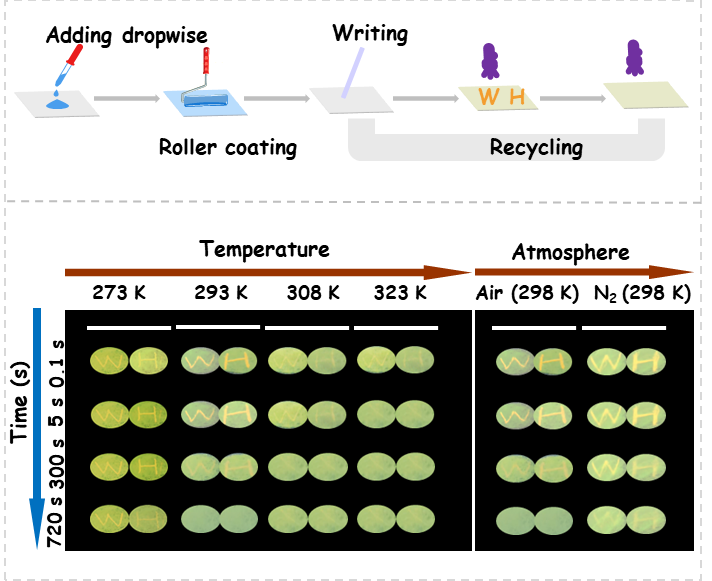


**Figure S58.** The self-recovery processes of IH-MPT film under mechanical force with the increased temperature.


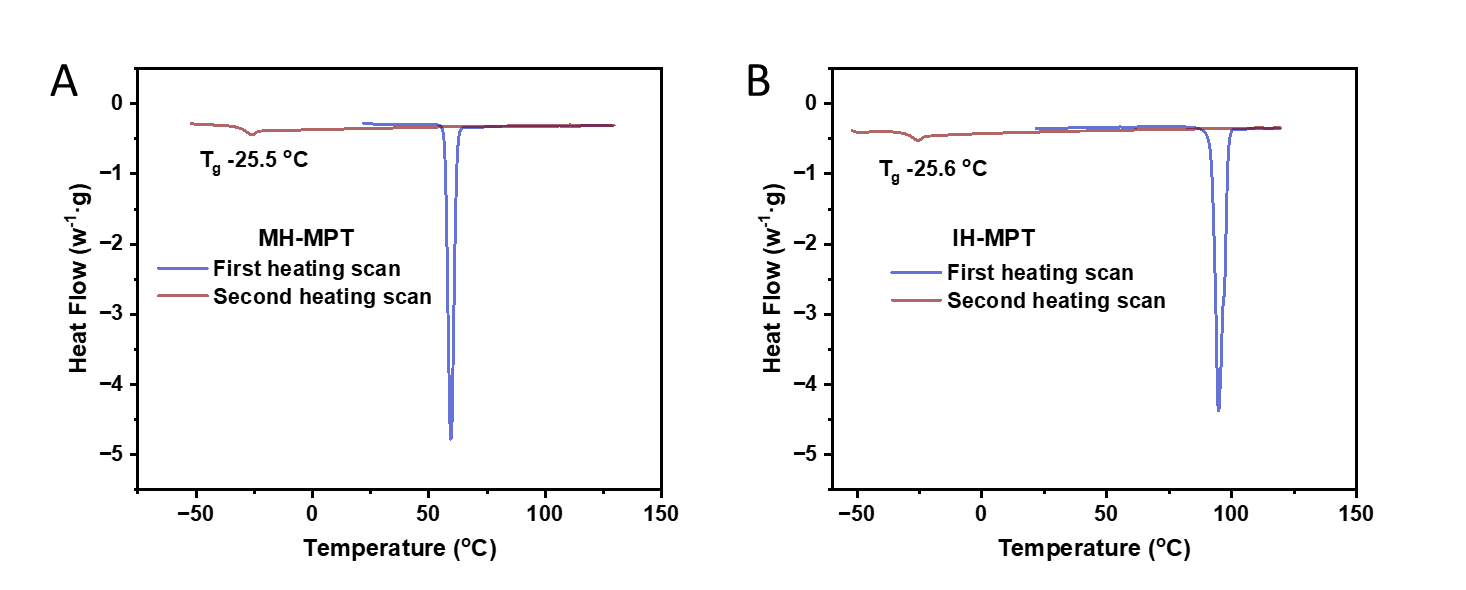


**Figure S59.** DSC thermograms of (a) MH-MPT and (b) IH-MPT by first heating scan (crystal state) and second heating scan (solidified state).


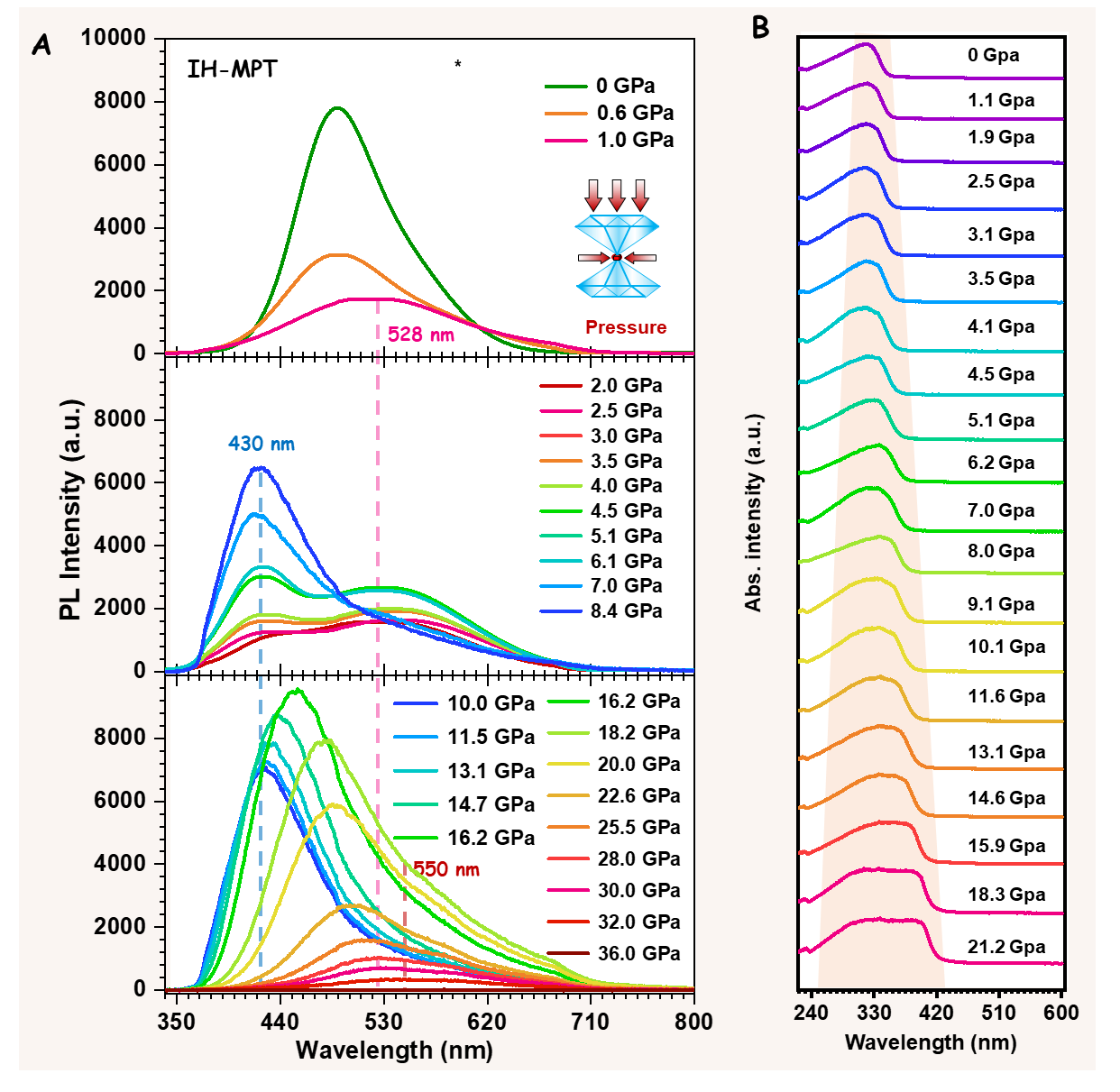


**Figure S60.** (A) Photoluminescence spectra of IH-MPT crystals under increased pressure from 0 GPa to 36.0 GPa. (B) Absorption spectra of IH-MPT crystals under increased pressure from 0 GPa to 21.2 GPa.


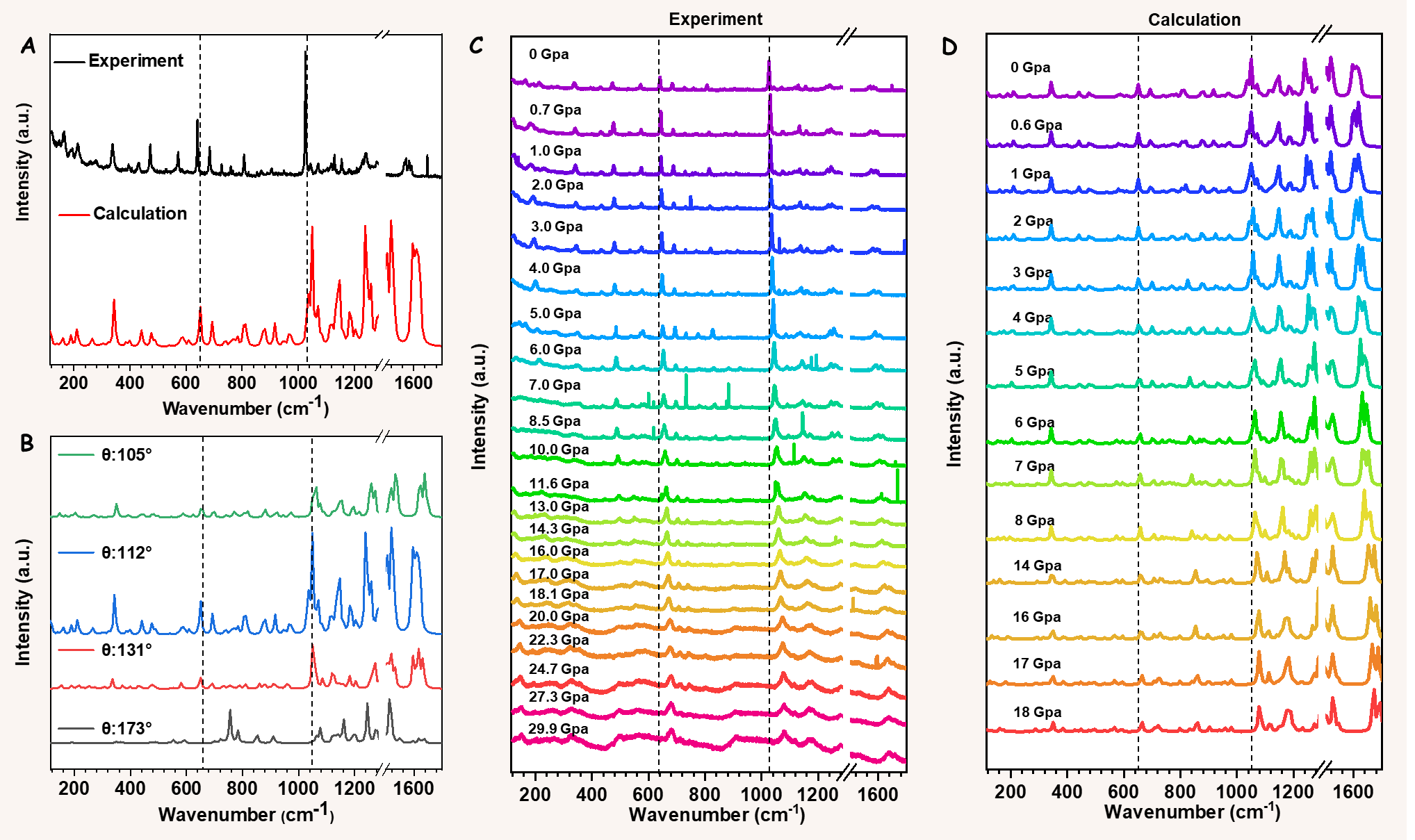


**Figure S61.** (A) Raman spectra of IH-MPT crystals. (B) Theoretical calculations: Angle dependent Raman spectroscopy. (C) Raman spectra of IH-MPT crystals under increased pressure from 0 GPa to 29.9 GPa (experiment). (D) Raman spectra of IH-MPT crystals under increased pressure from 0 GPa to 18 GPa (calculation).

**Figure S62.** Raman spectra of IH-MPT crystals under pressure from 0 GPa and R0 GPa (calculation)

**Figure S63.** Photoluminescence spectra of IH-MPT crystal under decreased pressure from R19.4 GPa to 0 GPa.

**Figure S64.** Absorption spectra of IH-MPT crystal under decreased pressure from R21.2 GPa to 0 GPa.

**Table S4** Potential surface scanning for HH-HPT and phenothiazine derivatives in Group1

| **HH-HPT** | | **MH-HPT** | | **IH-HPT** | | **MM-HPT** | | **IM-HPT** | |
| --- | --- | --- | --- | --- | --- | --- | --- | --- | --- |
| θ  (°) | E  (kcal•mol^-1^) | θ  (°) | E  (kcal•mol^-1^) | θ  (°) | E  (kcal•mol^-1^) | θ  (°) | E  (kcal•mol^-1^) | θ  (°) | E  (kcal•mol^-1^) |
| **178.0596256** | **-574575.0473** | **177.3553844** | **-599247.0234** | **170.3187752** | **-648583.7504** | **174.0194897** | **-623918.9558** | **172.8573986** | **-673255.694** |
| 169.2596256 | -574574.6301 | 168.5553844 | -599246.5447 | 162.3187752 | -648583.2348 | 165.6194897 | -623918.3283 | 164.5573986 | -673255.3235 |
| 160.4596256 | -574573.6466 | 159.7553844 | -599245.567 | 154.3187752 | -648582.523 | 157.2194897 | -623917.3304 | 156.2573986 | -673254.7292 |
| 151.6596256 | -574572.2349 | 150.9553844 | -599244.2464 | 146.3187752 | -648581.7219 | 148.8194897 | -623917.4453 | 147.9573986 | -673254.0334 |
| 142.8596256 | -574572.0628 | 142.1553844 | -599244.3829 | 138.3187752 | -648580.9027 | 140.4194897 | -623916.6817 | 139.6573986 | -673253.2939 |
| 134.0596256 | -574571.0785 | 133.3553844 | -599243.4775 | 130.3187752 | -648580.1062 | 132.0194897 | -623915.8548 | 131.3573986 | -673252.5362 |
| 125.2596256 | -574570.1296 | 124.5553844 | -599242.5693 | 122.3187752 | -648579.3697 | 123.6194897 | -623914.9623 | 123.0573986 | -673251.796 |
| 116.4596256 | -574569.2383 | 115.7553844 | -599241.6775 | 114.3187752 | -648578.7251 | 115.2194897 | -623914.0321 | 114.7573986 | -673251.6663 |
| 107.6596256 | -574568.4525 | 106.9553844 | -599240.8532 | 106.3187752 | -648578.659 | 106.8194897 | -623913.1564 | 106.4573986 | -673250.9137 |
| 98.85962556 | -574567.7961 | 98.15538442 | -599240.1546 | 98.31877518 | -648578.146 | 98.41948969 | -623912.4077 | 98.15739856 | -673250.3483 |
| **90.05962556** | **-574567.2016** | **89.35538442** | **-599239.5346** | **90.31877518** | **-648577.6887** | **90.01948969** | **-623911.7605** | **89.85739856** | **-673249.8799** |

Note: Red labels presented the initial conformations. Green labels presented the *ax* conformations.

**Table S5** **Potential surface scanning for phenothiazine derivatives in Group 2.**

| **HH-MPT** | | **HH-IPT** | | | | **MH-MPT** | | **IH-MPT** | |
| --- | --- | --- | --- | --- | --- | --- | --- | --- | --- |
| **Θ**  **(°)** | **E**  **(kcal•mol^-1^)** | **Θ**  **(°)** | **E**  **(kcal•mol^-1^)** | **Θ**  **(°)** | **E**  **(kcal•mol^-1^)** | **Θ**  **(°)** | **E**  **(kcal•mol^-1^)** | **Θ**  **(°)** | **E**  **(kcal•mol^-1^)** |
| **179.9471835** | **-599237.0137** | **179.7134452** | **-648569.3625** | **179.6113205** | **-648569.4262** | **176.4490936** | **-623897.5399** | **173.4382046** | **-673235.8089** |
| 178.5471835 | -599237.3694 | 177.0134452 | -648570.5093 | 177.1113205 | -648570.4726 | 172.6490936 | -623900.8844 | 166.6382046 | -673238.4742 |
| 177.1471835 | -599237.6925 | 174.3134452 | -648571.4465 | 174.6113205 | -648571.3491 | 168.8490936 | -623902.2827 | 159.8382046 | -673240.4038 |
| 175.7471835 | -599237.9826 | 171.6134452 | -648572.2622 | 172.1113205 | -648572.1202 | 165.0490936 | -623903.4372 | 153.0379461 | -673241.7026 |
| 174.3471835 | -599238.2393 | 168.9134452 | -648572.965 | 169.6113205 | -648572.7938 | 161.2490936 | -623904.3604 | 146.2379461 | -673242.4902 |
| 172.9471835 | -599238.4624 | 166.2134452 | -648573.5589 | 167.1113205 | -648573.373 | 157.4490936 | -623905.071 | 139.4379461 | -673242.8764 |
| 171.5471835 | -599238.6525 | 163.5134452 | -648574.05 | 164.6113205 | -648573.8621 | 153.6490936 | -623905.591 | 132.6379461 | -673242.9797 |
| 170.1471835 | -599238.8103 | 160.8134452 | -648574.4462 | 162.1113205 | -648574.267 | 149.8490936 | -623905.9456 | 125.8379461 | -673242.9474 |
| 168.7471835 | -599238.9369 | 158.1134452 | -648574.7544 | 159.6113205 | -648574.594 | 146.0490936 | -623906.1606 | 119.0379461 | -673243.0735 |
| 167.3471835 | -599239.0337 | 155.4134452 | -648574.9793 | 157.1113205 | -648574.8473 | **142.2490936** | **-623906.2595** | **112.2379461** | **-673243.4852** |
| **165.9471835** | **-599239.1021** | **152.7134452** | **-648575.128** | **154.6113205** | **-648575.0309** | 137.0490936 | -623906.2443 | 110.0379461 | -673243.5601 |
| 158.3471835 | -599239.038 | 146.4134452 | -648575.2481 | 148.1113205 | -648575.2411 | 131.8490936 | -623906.0882 | 107.8379461 | -673243.6063 |
| 150.7471835 | -599238.3767 | 140.1134452 | -648575.1518 | 141.6113205 | -648575.1897 | 126.6490936 | -623905.8202 | 105.6379461 | -673243.6221 |
| 143.1471835 | -599237.3149 | 133.8134452 | -648574.9155 | 135.1113205 | -648574.9729 | 121.4490936 | -623905.5999 | 103.4379461 | -673243.6006 |
| 135.5471835 | -599236.0416 | 127.5134452 | -648574.6061 | 128.6113205 | -648574.6612 | 116.2490936 | -623905.8198 | 101.2379461 | -673243.538 |
| 127.9471835 | -599234.6934 | 121.2134452 | -648574.3682 | 122.1113205 | -648574.3833 | 111.0490936 | -623905.9105 | 99.03794608 | -673243.4321 |
| 120.3471835 | -599233.4358 | 114.9134452 | -648574.1805 | 115.6113205 | -648574.2161 | 105.8490936 | -623905.8452 | 96.83794608 | -673243.2727 |
| 112.7471835 | -599232.3653 | 108.6134452 | -648573.5021 | 109.1113205 | -648573.5847 | 100.6490936 | -623905.5929 | 94.63794608 | -673243.0441 |
| 105.1471835 | -599231.3557 | 102.3134452 | -648571.8515 | 102.6113205 | -648571.9592 | 95.44909359 | -623905.0954 | 92.43794608 | -673242.7348 |
| 97.54718349 | -599230.3326 | 96.01344523 | -648568.7121 | 96.11132049 | -648568.7751 | **90.24909359** | **-623904.1912** | **90.23794608** | **-673242.33** |
| **89.94718349** | **-599228.999** | **89.71344523** | **-648571.7174** | **89.61132049** | **-648571.6976** |  |  |  |  |

Note: Red labels presented the initial conformations. Green labels presented the *ax/eq* conformations.

**Table S6 Potential surface scanning for phenothiazine derivatives in Group 3.**

| **MM-MPT** | | **IM-MPT** | | **II-MPT** | | **MM-IPT** | | **IM-IPT** | |
| --- | --- | --- | --- | --- | --- | --- | --- | --- | --- |
| **Θ**  **(°)** | **E**  **(kcal•mol^-1^)** | **Θ**  **(°)** | **E**  **(kcal•mol^-1^)** | **Θ**  **(°)** | **E**  **(kcal•mol^-1^)** | **Θ**  **(°)** | **E**  **(kcal•mol^-1^)** | **Θ**  **(°)** | **E**  **(kcal•mol^-1^)** |
| **173.599746** | **-648561.5853** | **179.5503064** | **-697892.2126** | **172.1634208** | **-747231.0663** | **179.7591818** | **-697876.7566** | **179.9316824** | **-747221.5069** |
| 166.799746 | -648566.3741 | 172.5503064 | -697896.8549 | 164.3644522 | -747236.8988 | 170.7591818 | -697882.4498 | 170.9316824 | -747230.7498 |
| 160.0000541 | -648569.7868 | 165.5503064 | -697901.8604 | 156.5644522 | -747241.6269 | 161.7591818 | -697887.1411 | 161.9316824 | -747238.7271 |
| 153.2000541 | -648572.4247 | 158.5503064 | -697905.652 | 148.7650434 | -747245.2445 | 152.7591818 | -697894.6729 | 152.9316824 | -747244.1065 |
| 146.4000541 | -648574.3769 | 151.5507512 | -697908.6497 | 140.9650434 | -747247.9106 | 143.7597709 | -697901.1899 | 143.9322831 | -747247.7232 |
| 139.6000541 | -648575.7396 | 151.5507512 | -697908.6497 | 133.1650434 | -747249.798 | 134.7597709 | -697906.3755 | 134.9322831 | -747250.0046 |
| 132.8000541 | -648576.5937 | 144.5507512 | -697910.9055 | 125.3650434 | -747251.0183 | 125.7597709 | -697910.4128 | 125.9322831 | -747251.2149 |
| 126.0000541 | -648577.0366 | 137.5507512 | -697912.51 | 117.5650434 | -747251.9162 | 116.7597709 | -697913.254 | 116.9322831 | -747250.3313 |
| 119.2000541 | -648576.8831 | 130.5507512 | -697913.5824 | 109.7650434 | -747252.9037 | 107.7597709 | -697914.27 | 107.9322831 | -747249.6045 |
| **112.4000541** | **-648577.4898** | 123.5507512 | -697914.1937 | **101.9650434** | **-747253.2659** | 98.75977087 | -697913.8753 | 98.9322831 | -747251.9148 |
| 110.2000541 | -648577.594 | 116.5507512 | -697914.6614 | 100.7650434 | -747253.2643 | **89.75977087** | **-697914.4195** | **89.9322831** | **-747252.6342** |
| 108.0000541 | -648577.6573 | **109.5507512** | **-697915.325** | 99.5650434 | -747253.2444 |  |  |  |  |
| 105.8000541 | -648577.6748 | 107.5507512 | -697915.4289 | 98.3650434 | -747253.2045 |  |  |  |  |
| 103.6000541 | -648577.645 | 105.5507512 | -697915.4924 | 97.1650434 | -747253.1432 |  |  |  |  |
| 101.4000541 | -648577.5701 | 103.5507512 | -697915.5137 | 95.9650434 | -747253.0597 |  |  |  |  |
| 99.20005412 | -648577.4434 | 101.5507512 | -697915.4948 | 94.7650434 | -747252.9531 |  |  |  |  |
| 97.00005412 | -648577.2585 | 99.55075124 | -697915.4362 | 93.5650434 | -747252.822 |  |  |  |  |
| 94.80005412 | -648577.0079 | 97.55075124 | -697915.3302 | 92.3650434 | -747252.6634 |  |  |  |  |
| 92.60005412 | -648576.6751 | 95.55075124 | -697915.1655 | 91.1650434 | -747252.4727 |  |  |  |  |
| **90.40005412** | **-648576.241** | 93.55075124 | -697914.934 | **89.9650434** | **-747252.2451** |  |  |  |  |
|  |  | 91.55075124 | -697914.6274 |  |  |  |  |  |  |
|  |  | **89.55075124** | **-697914.2278** |  |  |  |  |  |  |

Note: Red labels presented the initial conformations. Green labels presented the *ax/eq* conformations.

**References**

1. Gao, Y. et al. Expounding the relationship between molecular conformation and room-temperature phosphorescence property by deviation angle. *Journal of Physical Chemistry Letters* **13**, 3251-3260 (2022).
2. Huang, A. R. et al. Organic persistent RTP crystals: from brittle to flexible by tunable self-partitioned molecular packing. *Advanced Materials*. **35**, 2209166 (2023).
3. Gong, Y. B. et al. Partially controlling molecular packing to achieve off-on mechanochromism through ingenious molecular design. *Advanced Optical Materials*. **8**, 1902036 (2020).
4. Ward, J. S. et al.The interplay of thermally activated delayed fluorescence (TADF) and room temperature organic phosphorescence in sterically-constrained donor-acceptor charge-transfer molecules. *Chemical Communications*. **52**, 2612-2615 (2016).
5. Casselman, M. D. et al. Beyond the hammett effect: using strain to alter the landscape of electrochemical potentials. *ChemPhysChem*. **18**, 2142-2146 (2017).
