# Supplementary material for: Accurately adjusted phenothiazine conformations: reversible conformation transformation at room temperature and self-recoverable stimuli-responsive phosphorescence
Source: Light Sci Appl. 2025 Feb 26;14:99. doi: 10.1038/s41377-024-01716-7 (PMC11862005; doi:10.1038/s41377-024-01716-7)

checkCIF/PLATON report

You have not supplied any structure factors. As a result the full set of tests cannot be run.

THIS REPORT IS FOR GUIDANCE ONLY. IF USED AS PART OF A REVIEW PROCEDURE FOR PUBLICATION, IT SHOULD NOT REPLACE THE EXPERTISE OF AN EXPERIENCED CRYSTALLOGRAPHIC REFEREE.

No syntax errors found. CIF dictionary Interpreting this report

Datablock: y

```
Bond precision:  C-C = 0.0047 A         Wavelength=0.71073

Cell:            a=19.968(3)      b=7.7334(11)     c=8.3685(12)
                 alpha=90         beta=90          gamma=90
Temperature:     296 K

                Calculated                Reported
Volume          1292.3(3)                 1292.3(3)
Space group     P 21 21 21                P 21 21 21
Hall group      P 2ac 2ab                 P 2ac 2ab
Moiety formula  C15 H15 N S               C15 H15 N S
Sum formula     C15 H15 N S               C15 H15 N S
Mr              241.34                    241.34
Dx,g cm-3       1.240                     1.240
Z               4                         4
Mu (mm-1)       0.227                     0.227
F000            512.0                     512.0
F000'           512.65
h,k,lmax        24,9,10                   24,9,10
Nref            2544[ 1494]               2519
Tmin,Tmax       0.978,0.978               0.588,0.746
Tmin'           0.978

Correction method= # Reported T Limits: Tmin=0.588 Tmax=0.746
AbsCorr = NONE

Data completeness= 1.69/0.99      Theta(max)= 26.000

R(reflections)= 0.0464( 2147)     wR2(reflections)= 0.1004( 2519)

S = 1.157                 Npar= 215
```

The following ALERTS were generated. Each ALERT has the format
test-name_ALERT_alert-type_alert-level.
Click on the hyperlinks for more details of the test.

Alert level C

```
PLAT089_ALERT_3_C Poor Data / Parameter Ratio (Zmax < 18) ........       6.95 Note
PLAT241_ALERT_2_C High   'MainMol' Ueq as Compared to Neighbors of         S1 Check
PLAT242_ALERT_2_C Low    'MainMol' Ueq as Compared to Neighbors of        C13 Check
PLAT245_ALERT_2_C U(iso) H1        Smaller than U(eq) N1        by      0.013 Ang**2
PLAT340_ALERT_3_C Low Bond Precision on  C-C Bonds ...............    0.00473 Ang.
PLAT420_ALERT_2_C D-H Bond Without Acceptor  N1       --H1       .     Please Check
```

Alert level G

```
PLAT012_ALERT_1_G No       _shelx_res_checksum Found in CIF ......     Please Check
PLAT933_ALERT_2_G Number of OMIT Records in Embedded .res File ...          2 Note
PLAT941_ALERT_3_G Average HKL Measurement Multiplicity ...........        4.0 Low
```

```
  0 ALERT level A = Most likely a serious problem - resolve or explain
  0 ALERT level B = A potentially serious problem, consider carefully
  6 ALERT level C = Check. Ensure it is not caused by an omission or oversight
  3 ALERT level G = General information/check it is not something unexpected

  1 ALERT type 1 CIF construction/syntax error, inconsistent or missing data
  5 ALERT type 2 Indicator that the structure model may be wrong or deficient
  3 ALERT type 3 Indicator that the structure quality may be low
  0 ALERT type 4 Improvement, methodology, query or suggestion
  0 ALERT type 5 Informative message, check
```

It is advisable to attempt to resolve as many as possible of the alerts in all categories. Often the minor alerts point to easily fixed oversights, errors and omissions in your CIF or refinement strategy, so attention to these fine details can be worthwhile. In order to resolve some of the more serious problems it may be necessary to carry out additional measurements or structure refinements. However, the purpose of your study may justify the reported deviations and the more serious of these should normally be commented upon in the discussion or experimental section of a paper or in the "special_details" fields of the CIF. checkCIF was carefully designed to identify outliers and unusual parameters, but every test has its limitations and alerts that are not important in a particular case may appear. Conversely, the absence of alerts does not guarantee there are no aspects of the results needing attention. It is up to the individual to critically assess their own results and, if necessary, seek expert advice.

Publication of your CIF in IUCr journals

A basic structural check has been run on your CIF. These basic checks will be run on all CIFs submitted for publication in IUCr journals (*Acta Crystallographica*, *Journal of Applied Crystallography*, *Journal of Synchrotron Radiation*); however, if you intend to submit to *Acta Crystallographica Section C* or *E* or *IUCrData*, you should make sure that full publication checks are run on the final version of your CIF prior to submission.

Publication of your CIF in other journals

Please refer to the *Notes for Authors* of the relevant journal for any special instructions relating to CIF submission.

PLATON version of 22/03/2021; check.def file version of 19/03/2021

Datablock y - ellipsoid plot

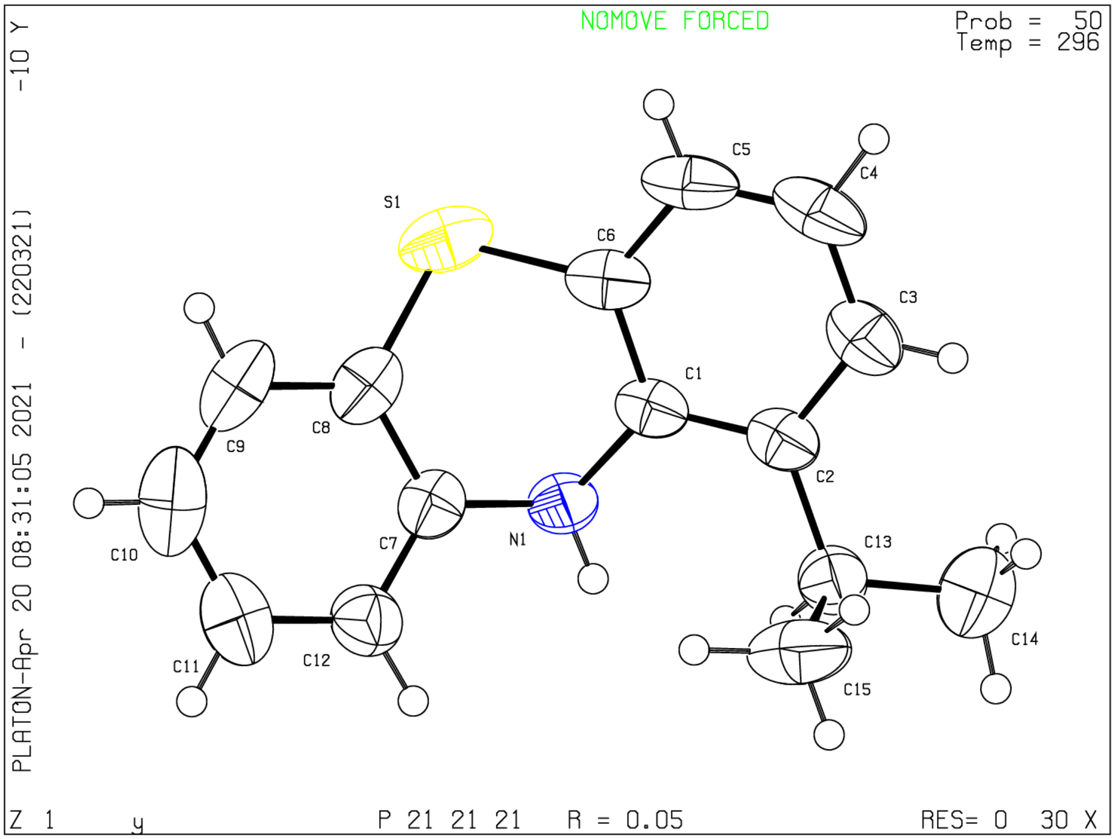

Supplement: Supplementary file 5 — Crystal informations [file 41377_2024_1716_MOESM5_ESM.zip › cif/IH-HPT checkcif.pdf]
