# Supplementary material for: Accurately adjusted phenothiazine conformations: reversible conformation transformation at room temperature and self-recoverable stimuli-responsive phosphorescence
Source: Light Sci Appl. 2025 Feb 26;14:99. doi: 10.1038/s41377-024-01716-7 (PMC11862005; doi:10.1038/s41377-024-01716-7)

checkCIF/PLATON report

You have not supplied any structure factors. As a result the full set of tests cannot be run.

THIS REPORT IS FOR GUIDANCE ONLY. IF USED AS PART OF A REVIEW PROCEDURE FOR PUBLICATION, IT SHOULD NOT REPLACE THE EXPERTISE OF AN EXPERIENCED CRYSTALLOGRAPHIC REFEREE.

No syntax errors found. CIF dictionary Interpreting this report

Datablock: 20220928-gy6-1_auto

```
Bond precision:   C-C = 0.0040 A          Wavelength=1.54184

Cell:               a=8.1235(3)      b=8.4076(3)      c=19.8254(7)
                    alpha=90         beta=90          gamma=90
Temperature:        301 K

                 Calculated                       Reported
Volume           1354.06(8)                       1354.06(8)
Space group      P 21 21 21                       P 21 21 21
Hall group       P 2ac 2ab                        P 2ac 2ab
Moiety formula   C16 H17 N S                      C16 H17 N S
Sum formula      C16 H17 N S                      C16 H17 N S
Mr               255.37                           255.36
Dx,g cm-3        1.253                            1.253
Z                4                                4
Mu (mm-1)        1.947                            1.947
F000             544.0                            544.0
F000'            546.55
h,k,lmax         10,10,24                         10,10,24
Nref             2826[ 1649]                      2494
Tmin,Tmax        0.823,0.823                      0.511,1.000
Tmin'            0.823

Correction method= # Reported T Limits: Tmin=0.511 Tmax=1.000
AbsCorr = MULTI-SCAN

Data completeness= 1.51/0.88       Theta(max)= 76.069

R(reflections)= 0.0527( 2417)                          wR2(reflections)=
                                                       0.1292( 2494)
S = 1.099                    Npar= 166
```

The following ALERTS were generated. Each ALERT has the format
test-name_ALERT_alert-type_alert-level.
Click on the hyperlinks for more details of the test.

Alert level C

```
PLAT242_ALERT_2_C Low    'MainMol' Ueq as Compared to Neighbors of        C14 Check
PLAT414_ALERT_2_C Short Intra D-H..H-X       H1       ..H14      .       1.98 Ang.
                                                       x,y,z  =      1_555 Check
PLAT420_ALERT_2_C D-H Bond Without Acceptor  N1       --H1       .     Please Check
PLAT790_ALERT_4_C Centre of Gravity not Within Unit Cell: Resd.  #          1 Note
              C16 H17 N S
```

Alert level G

```
PLAT007_ALERT_5_G Number of Unrefined Donor-H Atoms .............           1 Report
PLAT941_ALERT_3_G Average HKL Measurement Multiplicity ..........         3.8 Low
```

0 **ALERT level A** = Most likely a serious problem - resolve or explain
0 **ALERT level B** = A potentially serious problem, consider carefully
4 **ALERT level C** = Check. Ensure it is not caused by an omission or oversight
2 **ALERT level G** = General information/check it is not something unexpected

0 ALERT type 1 CIF construction/syntax error, inconsistent or missing data
3 ALERT type 2 Indicator that the structure model may be wrong or deficient
1 ALERT type 3 Indicator that the structure quality may be low
1 ALERT type 4 Improvement, methodology, query or suggestion
1 ALERT type 5 Informative message, check

It is advisable to attempt to resolve as many as possible of the alerts in all categories. Often the minor alerts point to easily fixed oversights, errors and omissions in your CIF or refinement strategy, so attention to these fine details can be worthwhile. In order to resolve some of the more serious problems it may be necessary to carry out additional measurements or structure refinements. However, the purpose of your study may justify the reported deviations and the more serious of these should normally be commented upon in the discussion or experimental section of a paper or in the "special_details" fields of the CIF. checkCIF was carefully designed to identify outliers and unusual parameters, but every test has its limitations and alerts that are not important in a particular case may appear. Conversely, the absence of alerts does not guarantee there are no aspects of the results needing attention. It is up to the individual to critically assess their own results and, if necessary, seek expert advice.

Publication of your CIF in IUCr journals

A basic structural check has been run on your CIF. These basic checks will be run on all CIFs submitted for publication in IUCr journals (*Acta Crystallographica*, *Journal of Applied Crystallography*, *Journal of Synchrotron Radiation*); however, if you intend to submit to *Acta Crystallographica Section C* or *E* or *IUCrData*, you should make sure that full publication checks are run on the final version of your CIF prior to submission.

Publication of your CIF in other journals

Please refer to the *Notes for Authors* of the relevant journal for any special instructions relating to CIF submission.

PLATON version of 12/09/2022; check.def file version of 09/08/2022

Datablock 20220928-gy6-1_auto - ellipsoid plot

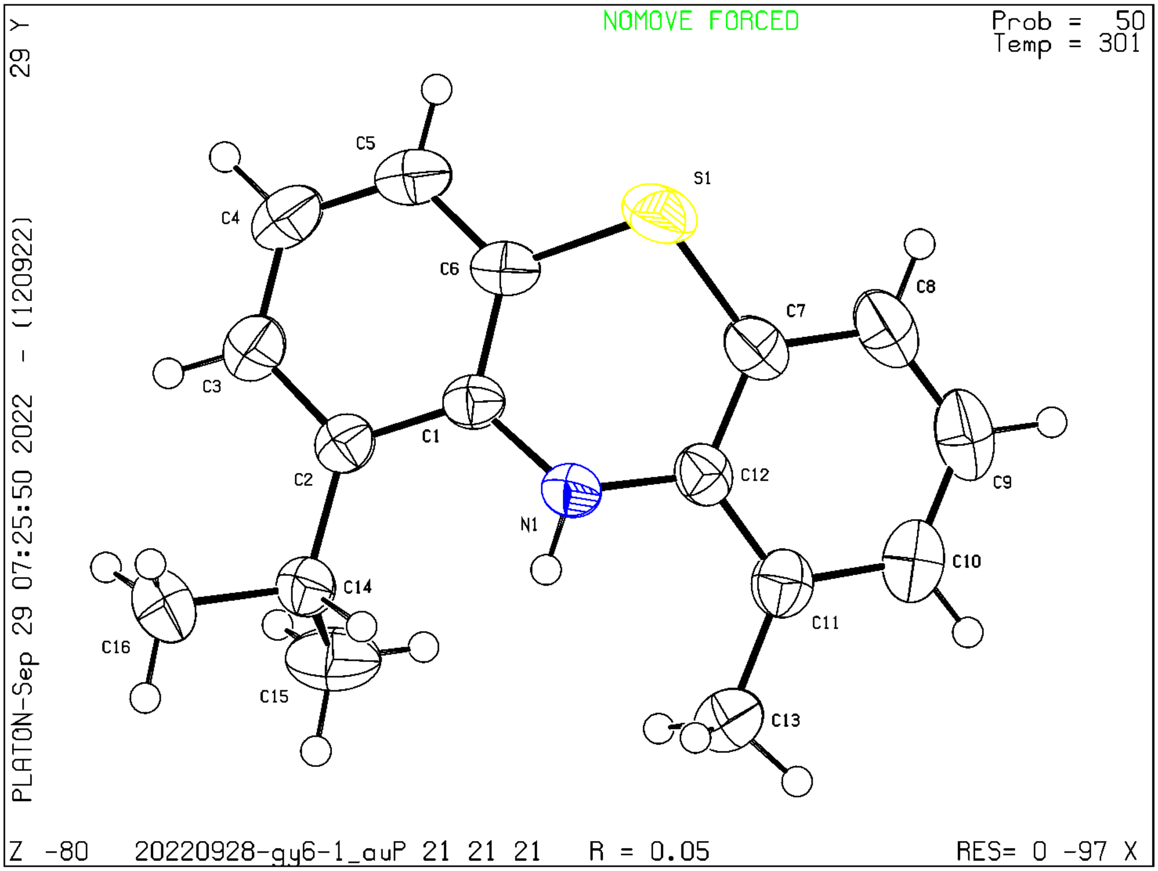

Supplement: Supplementary file 5 — Crystal informations [file 41377_2024_1716_MOESM5_ESM.zip › cif/IM-HPT checkcif.pdf]
