# Supplementary material for: Accurately adjusted phenothiazine conformations: reversible conformation transformation at room temperature and self-recoverable stimuli-responsive phosphorescence
Source: Light Sci Appl. 2025 Feb 26;14:99. doi: 10.1038/s41377-024-01716-7 (PMC11862005; doi:10.1038/s41377-024-01716-7)

checkCIF/PLATON report

You have not supplied any structure factors. As a result the full set of tests cannot be run.

THIS REPORT IS FOR GUIDANCE ONLY. IF USED AS PART OF A REVIEW PROCEDURE FOR PUBLICATION, IT SHOULD NOT REPLACE THE EXPERTISE OF AN EXPERIENCED CRYSTALLOGRAPHIC REFEREE.

No syntax errors found. CIF dictionary Interpreting this report

Datablock: mo_210918C_0m

```
Bond precision:   C-C = 0.0047 A          Wavelength=0.71073

Cell:              a=11.694(2)     b=8.2136(15)        c=15.796(3)
                   alpha=90        beta=99.276(3)      gamma=90
Temperature:       296 K

                 Calculated                 Reported
Volume           1497.4(5)                  1497.4(5)
Space group      P 21/c                     P 1 21/c 1
Hall group       -P 2ybc                    -P 2ybc
Moiety formula   C17 H19 N S                C17 H19 N S
Sum formula      C17 H19 N S                C17 H19 N S
Mr               269.39                     269.41
Dx,g cm-3        1.195                      1.195
Z                4                          4
Mu (mm-1)        0.203                      0.203
F000             576.0                      576.7
F000'            576.67
h,k,lmax         13,9,18                    13,9,18
Nref             2630                       2629
Tmin,Tmax        0.980,0.980                0.654,0.746
Tmin'            0.980

Correction method= # Reported T Limits: Tmin=0.654 Tmax=0.746
AbsCorr = NONE

Data completeness= 1.000           Theta(max)= 25.020

                                                     wR2(reflections)=
R(reflections)= 0.0708( 2251)                        0.2005( 2629)

S = 1.102                    Npar= 248
```

The following ALERTS were generated. Each ALERT has the format
test-name_ALERT_alert-type_alert-level.
Click on the hyperlinks for more details of the test.

Alert level C

```
SHFSU01_ALERT_2_C  The absolute value of parameter shift to su ratio > 0.05
            Absolute value of the parameter shift to su ratio given   0.055
            Additional refinement cycles may be required.
PLAT241_ALERT_2_C High   'MainMol' Ueq as Compared to Neighbors of         S1 Check
PLAT245_ALERT_2_C U(iso) H16B      Smaller than U(eq) C16       by      0.014 Ang**2
PLAT340_ALERT_3_C Low Bond Precision on  C-C Bonds ...............    0.00475 Ang.
PLAT350_ALERT_3_C Short  C-H (X0.96,N1.08A)  C14      - H14B     .       0.84 Ang.
PLAT351_ALERT_3_C Long   C-H (X0.96,N1.08A)  C16      - H16B     .       1.16 Ang.
PLAT911_ALERT_3_C Missing FCF Refl Between Thmin & STh/L=    0.595          4 Report
PLAT977_ALERT_2_C Check Negative Difference Density on H15              -0.41 eA-3
```

Alert level G

```
PLAT002_ALERT_2_G Number of Distance or Angle Restraints on AtSite          4 Note
PLAT072_ALERT_2_G SHELXL First  Parameter in WGHT  Unusually Large       0.12 Report
PLAT172_ALERT_4_G The CIF-Embedded .res File Contains DFIX Records          2 Report
PLAT769_ALERT_4_G CIF Embedded explicitly supplied scattering data     Please Note
PLAT860_ALERT_3_G Number of Least-Squares Restraints .............          2 Note
PLAT909_ALERT_3_G Percentage of I>2sig(I) Data at Theta(Max) Still        69% Note
PLAT941_ALERT_3_G Average HKL Measurement Multiplicity ..........         3.7 Low
PLAT960_ALERT_3_G Number of Intensities with I  <   - 2*sig(I) ...         17 Check
PLAT978_ALERT_2_G Number C-C Bonds with Positive Residual Density.          0 Info
PLAT983_ALERT_1_G The  S-f"=    0.1244 Deviates from  IT-Value =       0.1234 Check
```

```
  0 ALERT level A = Most likely a serious problem - resolve or explain
  0 ALERT level B = A potentially serious problem, consider carefully
  8 ALERT level C = Check. Ensure it is not caused by an omission or oversight
 10 ALERT level G = General information/check it is not something unexpected

  1 ALERT type 1 CIF construction/syntax error, inconsistent or missing data
  7 ALERT type 2 Indicator that the structure model may be wrong or deficient
  8 ALERT type 3 Indicator that the structure quality may be low
  2 ALERT type 4 Improvement, methodology, query or suggestion
  0 ALERT type 5 Informative message, check
```

It is advisable to attempt to resolve as many as possible of the alerts in all categories. Often the minor alerts point to easily fixed oversights, errors and omissions in your CIF or refinement strategy, so attention to these fine details can be worthwhile. In order to resolve some of the more serious problems it may be necessary to carry out additional measurements or structure refinements. However, the purpose of your study may justify the reported deviations and the more serious of these should normally be commented upon in the discussion or experimental section of a paper or in the "special_details" fields of the CIF. checkCIF was carefully designed to identify outliers and unusual parameters, but every test has its limitations and alerts that are not important in a particular case may appear. Conversely, the absence of alerts does not guarantee there are no aspects of the results needing attention. It is up to the individual to critically assess their own results and, if necessary, seek expert advice.

Publication of your CIF in IUCr journals

A basic structural check has been run on your CIF. These basic checks will be run on all CIFs submitted for publication in IUCr journals (*Acta Crystallographica*, *Journal of Applied Crystallography*, *Journal of Synchrotron Radiation*); however, if you intend to submit to *Acta Crystallographica Section C* or *E* or *IUCrData*, you should make sure that full publication checks are run on the final version of your CIF prior to submission.

Publication of your CIF in other journals

Please refer to the *Notes for Authors* of the relevant journal for any special instructions relating to CIF submission.

PLATON version of 13/07/2021; check.def file version of 13/07/2021

Datablock mo_210918C_0m - ellipsoid plot

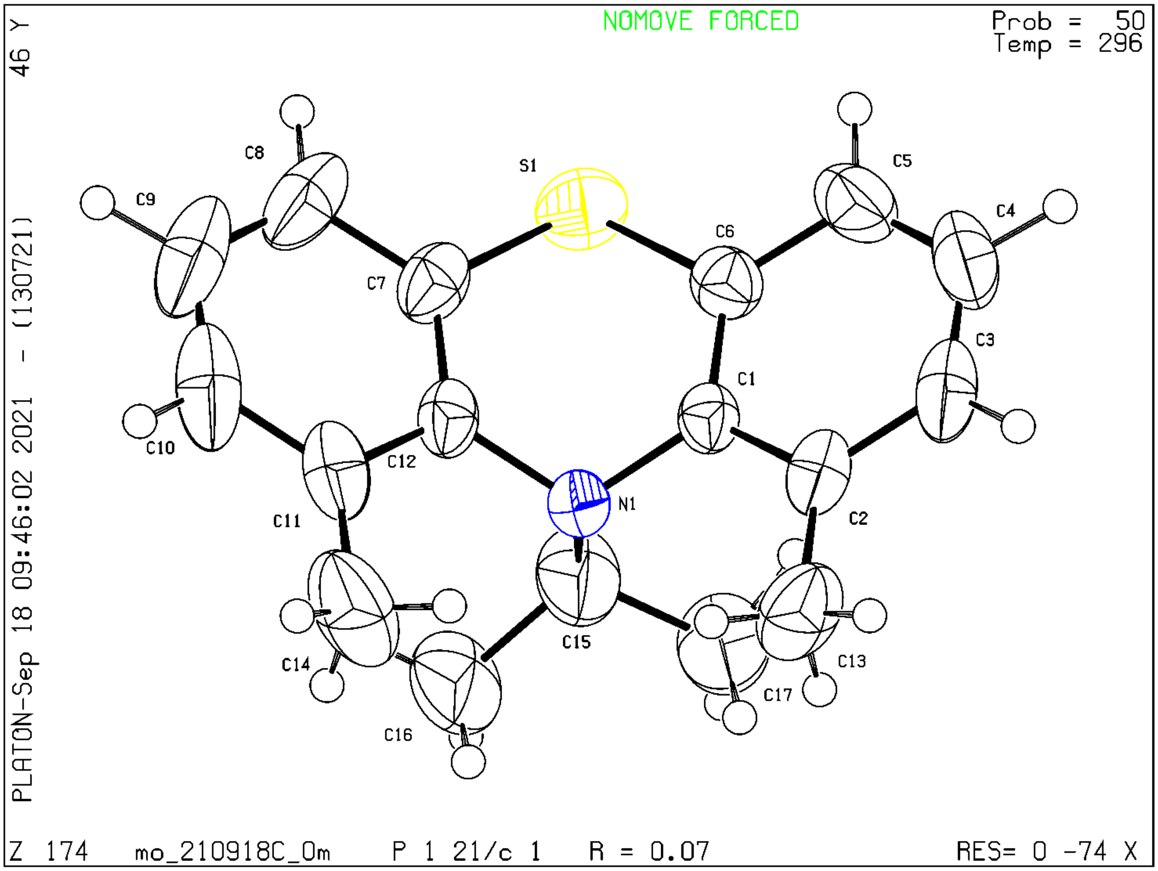

Supplement: Supplementary file 5 — Crystal informations [file 41377_2024_1716_MOESM5_ESM.zip › cif/MM-IPT checkcif.pdf]
