# Supplementary material for: Accurately adjusted phenothiazine conformations: reversible conformation transformation at room temperature and self-recoverable stimuli-responsive phosphorescence
Source: Light Sci Appl. 2025 Feb 26;14:99. doi: 10.1038/s41377-024-01716-7 (PMC11862005; doi:10.1038/s41377-024-01716-7)
Supplement: Supplementary file 6 — copyright [file 41377_2024_1716_MOESM6_ESM.pdf]

文章保密与版权转让证明

承　诺　书

此文章不涉密且不存在造假、抄袭、一稿多投等学术不端行为，特此承诺。

第一（通讯）作者签字：高原

2024 年 12 月 4 日

《Light: Science & Applications》编辑部：

我单位 高原、袁文涛、李相钦、黄阳娣、樊瑶媛、李振森、王凯、邹勃、李倩倩、李振 作者（需按正式发表文章署名顺序，填写全部作者姓名）为你刊撰写的文章（题目：Accurately Adjusted phenothiazine conformations, Reversible Conformation Transformation at Room Temperature and Self-Recoverable Stimuli-responsive phosphorescence）；经审查，未发现该文章存在涉密内容和造假、抄袭、一稿多投等学术不端现象。该文章若存在涉密内容和造假、抄袭、一稿多投等学术不端问题，《Light: Science & Applications》编辑部无需承担任何责任。该文章一经录用，其数字化复制权、发行权、汇编权及信息网络传播权将转让予《Light: Science & Applications》编辑部。

导师（课题负责人）签字 李振

单位盖章 武汉大学化学与分子科学学院

2024 年 12 月 4 日
